# Effective refolding of a cysteine rich glycoside hydrolase family 19 recombinant chitinase from *Streptomyces griseus* by reverse dilution and affinity chromatography

**DOI:** 10.1371/journal.pone.0241074

**Published:** 2020-10-22

**Authors:** Ayokunmi Omolola Oyeleye, Siti Faridah Mohd Yusoff, Izzah Nadiah Abd Rahim, Adam Thean Chor Leow, Noor Baity Saidi, Yahaya M. Normi

**Affiliations:** 1 Department of Cell and Molecular Biology, Faculty of Biotechnology and Biomolecular Science, Universiti Putra Malaysia, Serdang, Selangor, Malaysia; 2 Enzyme and Microbial Technology Research Center, Faculty of Biotechnology and Biomolecular Sciences, Universiti Putra Malaysia, Selangor, Malaysia; Instituto Butantan, BRAZIL

## Abstract

Conventional refolding methods are associated with low yields due to misfolding and high aggregation rates or very dilute proteins. In this study, we describe the optimization of the conventional methods of reverse dilution and affinity chromatography for obtaining high yields of a cysteine rich recombinant glycoside hydrolase family 19 chitinase from *Streptomyces griseus* HUT6037 (SgChiC). SgChiC is a potential biocontrol agent and a reference enzyme in the study and development of chitinases for various applications. The overexpression of SgChiC was previously achieved by periplasmic localization from where it was extracted by osmotic shock and then purified by hydroxyapatite column chromatography. In the present study, the successful refolding and recovery of recombinant SgChiC (r-SgChiC) from inclusion bodies (IB) by reverse dilution and column chromatography methods is respectively described. Approximately 8 mg of r-SgChiC was obtained from each method with specific activities of 28 and 52 U/mg respectively. These yields are comparable to that obtained from a 1 L culture volume of the same protein isolated from the periplasmic space of *E*. *coli* BL21 (DE3) as described in previous studies. The higher yields obtained are attributed to the successful suppression of aggregation by a stepwise reduction of denaturant from high, to intermediate, and finally to low concentrations. These methods are straight forward, requiring the use of fewer refolding agents compared with previously described refolding methods. They can be applied to the refolding of other cysteine rich proteins expressed as inclusion bodies to obtain high yields of actively folded proteins. This is the first report on the recovery of actively folded SgChiC from inclusion bodies.

## Introduction

Inclusion bodies (IB) formed during protein expression in *Escherichia coli* are a frequently encountered phenomenon with up to 70% of target recombinant proteins reportedly ending up as IB thereby impeding the production and study of such enzymes [1–3]. IBs have some advantages which include a high level of purity since they contain fewer background (host) proteins co-expressed with the protein of interest, thus requiring less purification steps [2,4]; they are less exposed to degradation by proteases [4] and harsh extraction procedures like sonication and high pressure cell disruption with a French press [5]; and can be conveniently stored in lyophilized or moist states or other conditions for extended periods prior to future refolding activities [5].

The recovery of proteins from IB is typically carried out by a combination of chemical and physical methods. Conventionally, IBs are first solubilized in high concentrations of chaotropic agents and then refolded under non-denaturing conditions by one of three methods; dialysis, dilution or column chromatography techniques. These methods have been reviewed elsewhere [4,6–9]. All three methods generally involve washing, denaturation and refolding steps. The washing and denaturation steps may require the use of high concentrations of detergents such as Triton X-100 [10], sodium N-lauroyl sarcosine (sarcosyl) [11] sodium dodecyl sulphate (SDS) [12] and chaotropic agents such as urea [1,13] and guanidine hydrochloride (Gdn-HCl). Refolding solubilized proteins thus requires the removal or optimum reduction of the denaturants by introducing a buffer with little or no chaotropic agents or detergents. However, this is often associated with loss in protein yield resulting from aggregation especially when proteins are at medium to high concentrations [9]. At such concentrations, non-specific hydrophobic interactions between folding intermediates take place causing molecules to collapse into aggregates [2]. In other words, the success of several refolding processes relies largely on low protein concentrations, wherein protein molecules are kept as widely dispersed as possible [14]. Such low concentrations are only suitable for limited applications in which a small amount of starting material is processed at a time [2,8]. Additionally, proteins with reduced cysteines are highly prone to aggregation which arise as a result of irreversible mispairing of free cysteines during refolding [15]. In order to circumvent the problem of aggregation and protein loss, the use of high hydrostatic pressure (HHP) has been applied in solubilizing and refolding of some IBs [15–20]. HHP in the range of 1–3 kbar for about 90 mins, in combination with mild concentrations of chaotropes or alkaline pH have been reported to enhance dissociation of oligomers and disaggregation of intermolecular hydrophobic and electrostatic interactions [15,21]. Refolding is then promoted at low pressure of about 0.4 bar for up to 15 hrs [20,22]. While several successes have been reported with the use of HHP, the availability of equipment might be a major limitation to its immediate application for any refolding process. Also, just like other refolding methods, optimal conditions required for successful refolding still requires significant screening and trials [23,24].

In this study, a stepwise reduction in denaturant concentration during refolding by reverse dilution (RD) and on-column (OnC) affinity chromatography is demonstrated while optimizing refolding conditions for obtaining higher yields of a recombinant chitinase from *Streptomyces griseus* HUT6037.

*Streptomyces griseus* HUT6037 chitinase (SgChiC) is the most studied non-plant glycoside hydrolase family 19 chitinase obtained from bacteria. It is a modular enzyme [25,26] that consists of an N-terminal chitin binding domain (CBD) belonging to the cellulose binding module family 5 (CBM-5) [29] of bacterial origin, and a C-terminal alpha helix-rich catalytic domain (CatD) having sequence and structural similarities with plant chitinases [27–29]. The CBD with 52 amino acid residues (Ala 30 –Gly 81) [27] contains 2 cysteine residues while the CatD (Gly 90 –Cys 294) contains 4 cysteine residues; altogether forming 3 disulphide bonds in total. Both domains are connected by a flexible linker with the sequence TGGEGPGGNN [26]. Its discovery as the first non-plant GH family 19 chitinase pioneered the research into other non-plant sources from the same family [25,30]. Currently, its 3-dimensional (3D) structure is one of only 2 bacterial GH family 19 chitinases available in the PDB. Hence, it is a source of valuable structural and biochemical information, and in fact a reference enzyme in the study, discovery and engineering of chitinases with novel or improved characteristics. Apart from its significance in research, SgChiC has antifungal properties and is of potential application in the biocontrol of fungal phytopathogens [29–31]. In previous studies, the expression of SgChiC appeared to be difficult with low or no expression after trials with some *E*. *coli* hosts. Subsequently, the expression construct was changed and inserted into a pET12-a(+) vector from where its expression was directed into the periplasmic space and then extracted by the cold osmotic shock method [29,32]. In this study as well, the poor expression of a C-terminal 6-Histidine tagged r-SgChiC in the soluble cytoplasmic extracts of *Escherichia coli* BL21(DE3) posed a major challenge to downstream studies and application because it was exclusively deposited as inclusion bodies (IB). This necessitated the development of a refolding protocol towards obtaining high yields of actively folded r-SgChiC. The successful refolding of r-SgChiC was carried out bearing in mind its propensity to be misfolded due to its richness in cysteines. Commercially synthesized *SgchiC* was cloned, expressed and recovered from its unfolded state by reverse dilution and affinity chromatography respectively. While reports on refolding of proteins by these methods are few due to the associated loss in protein yield, they were optimized as described here to obtain appreciable yields of r-SgChiC. Hence, the successful application of reverse dilution and affinity chromatography in this study reveals their potential as efficient methods in obtaining well folded proteins from IBs. They are simple and do not require specialized equipment other than those used in routine protein production and purification steps. Also, very few chemicals are needed for solubilizing, reducing and refolding viz; guanidinium hydrochloride (Gdn-HCl), dithiothreitol (DTT), urea and L-arginine dissolved in suitable buffers. The simplicity of these methods renders them suitable for any initial refolding protocol for other proteins with a high propensity for IB formation or aggregation.

## Materials and methods

### Plasmids, host strains and chemicals

pET22-b(+) (Novagen) was used as the cloning and expression vector throughout the study. The vector is equipped with an N-terminal *pel*B coding sequence for potential periplasmic localization and an optional C-terminal His-Tag^®^. However, the *pel*B sequence was excluded (as described in the section on cloning) to prevent the translocation of r-SgChiC into the periplasmic space. *Escherichia coli* DH5α and BL21(DE3) (Invitrogen, USA) were used for cloning and expression respectively. All chemicals were of high grade and they include, guanidine hydrochloride (Gdn-HCl) and DL-Dithiothreitol (DTT) from Calbiochem (Germany). Urea and L-arginine were purchased from Merck (Germany) while reduced and oxidized glutathione were from Sigma (USA).

### Genes and oligonucleotides

The protein sequence of *S*. *griseus* HUT6037 ChiC (SgChiC) was retrieved from the Protein Data Bank (PDB), back translated to nucleotide sequence using the EMBOSS Backtranseq web based tool (www.ebi.ac.uk/Tools/st/emboss_backtranseq) and verified by alignment with the complete genome of *Streptomyces griseus* subsp. *griseus* NBRC 13350 (retrieved from NCBI Gen Bank). Codon optimization was performed on the verified sequence for expression in *Escherichia coli* B series using the codon usage table at http://www.kazusa.or.jp/codon/ accessed through the online tool at www.atgme.org. The restriction endonuclease sites *Nco*I and *Bam*HI, were included in the sequence on the 5’ and 3’ ends respectively. The codon optimized 795 kbp sequence (covering the amino acid sequence from Ala30-Cys295 without signal peptide) coding for *SgChiC* was commercially synthesized and delivered as pUC119 vector inserts (Integrated DNA Technologies (IDT) Inc.).

### Cloning of synthetic *SgChiC* gene for expression

The plasmid designated pUC119::*SgchiC* was first re-transformed into competent *E*. *coli* DH5α cells by the heat shock procedure. pUC119::*SgchiC* plasmids were isolated following a plasmid mini prep from an overnight culture of a single colony of successfully transformed cells. The *SgchiC* inserts were isolated by PCR amplification using the following designed primers with *Nde*I and *Xho*I restriction sites (RE) incorporated on the forward and reverse primers respectively: 5’-ATACATATGGCGACCTGCGCGCGACCG-3’ and 5’-GGTCTCGAGGCAGCTCAGATTCGGGC-3’. The underlined sequences are the respective RE sites. The amplified inserts were restricted with *Nde*I and *Xho*I and then ligated using T4 DNA ligase (NEB, USA) to pET22-b(+) vector which was similarly digested with the same REs to exclude the *pel*B coding sequence and prevent subsequent deposition of r-SgChiC in the periplasmic space. The ligated product (now designated pET22-b(+)::*SgchiC*) was subsequently transformed into *E*. *coli* BL21(DE3) expression host. Successful clones harbouring pET22-b(+)::*SgchiC* were analyzed on 0.8% agarose gel and further confirmed by Sanger sequencing (Apical Scientific Sdn. Bhd.).

### Growth conditions and isolation of inclusion bodies

A single colony of recombinant *E*. *coli* BL21(DE3) harboring pET22-b(+)::*SgchiC* was inoculated in 10 mL Luria Bertani (LB) broth and cultivated at 37°C and 200 rpm for 18 hours. 10 mL of the culture was used to inoculate a 1 L LB broth supplemented with 100 μg/mL of ampicillin. Cultures were maintained at 37°C until cells were at mid log phase and attained an A_600_ of ~0.6, monitored with a Varian Cary 50 Bio UV-Vis spectrophotometer. Expression of recombinant r-SgChiC was then induced with IPTG to a final concentration of 0.2 mM and the culture was further cultivated for 2 hours at 30°C. Cultures were harvested by centrifugation at 8000 rpm (F-34-6-38 rotor in a 5804R Eppendorf centrifuge) for 10 min and pellets were either stored at -20°C or washed immediately. Cell pellets were re-suspended in wash buffer (WB) (50 mM Tris-HCl pH 8.0, supplemented with 0.3 M NaCl) and then disrupted by sonication for 3 minutes with 30 secs pulse at 40% power. The resulting lysate was separated by centrifugation at 8000 rpm and 4°C for 20 minutes. The presence of the target protein in the insoluble fraction was subsequently analyzed by SDS-PAGE.

### Solubilization of inclusion bodies

IBs obtained from 500 mL or 1.5 g of disrupted pellets from a 1 L culture were divided and each re-suspended in two 50 mL tubes with 15 mL of wash buffer (WB) (50 mM Tris-HCl, pH 8.0, 0.3 M NaCl) supplemented with 1 M urea. Resuspension was performed by continuous pipetting until homogeneity was attained. Washed pellets were then harvested by centrifugation at 8000 rpm for 20 minutes at 4°C. The washing step was repeated a second time and supernatants were analyzed by SDS-PAGE to confirm that the protein was retained in the insoluble fraction before discarding. Subsequently, washed pellets were pooled and solubilized by re-suspension in 5 mL of binding buffer (BB) containing 20 mM Tris-HCl (pH 8), 0.3 M NaCl, 20 mM imidazole and 6 M Gdn-HCl. Pellets were solubilized by continuous pipetting and occasional vortex until the suspension appeared homogenous. Solubilized IBs were then separated from other insoluble material by immediate centrifugation to enhance recovery of the solubilized fraction from the remaining viscous cellular components at 8000 rpm and 4°C for 20 minutes. The solubilized protein was then reduced with 10 mM DTT and incubated for 2 hours at room temperature or 18 hours at 4°C before being subsequently refolded by reverse dilution or on-column refolding respectively.

### Refolding by reverse dilution

In order to obtain purer protein, the solubilized and reduced r-SgChiC (prior to reverse dilution) was applied to a Ni-NTA column equilibrated with 5 CV of BB (20 mM Tris-HCl, pH 8.0, 20 mM imidazole, 0.3 M NaCl and 6 M Gdn-HCl). The bound protein was washed with solubilizing buffer (SB) (20 mM Tris-HCl pH8, 20 mM imidazole, 0.3 M NaCl and 6 M urea) and then eluted on a gradient with 20 mL of elution buffer (EB) (20 mM Tris-HCl pH 8, 0.3 M NaCl, 0.6 M imidazole) supplemented with 6 M urea at a flow rate of 0.2 mL/min. Peak fractions were pooled making up to 10 mL of purified and denatured protein. Purified r-SgChiC was transferred into a beaker and subsequently refolded by reverse dilution as follows: The beaker containing purified and solubilized r-SgChiC was placed on ice and on a magnetic stirrer. The protein solution was first diluted in the ratio 1:1 by the addition of refolding buffer (RB) (20 mM Tris-HCl and 0.3 M NaCl, pH 8) supplemented with 2 M urea with the use of a peristaltic pump at a flow rate of 0.5 mL/min. This was followed by a second dilution step similarly in the same ratio as above but with RB containing 1 M urea. In the absence of precipitates, a third and fourth dilution step with RB supplemented with 1 M urea and dialysis buffer (DB) (20 mM K_2_HPO_4_/KH_2_PO_4_ pH 8.0, 0.05 M NaCl, 0.02 M arginine) supplemented with 0.2 M arginine, in the ratios of 1:1 and 2:1 were carried out respectively. The dilution ratios refer to the ratio of volume of sample just before dilution to the volume of buffer to be used. Subsequently, the diluted, partially refolded r-SgChiC was subjected to dialysis. Dialysis was carried out firstly for 16 hours in dialysis buffer (20 mM K_2_HPO_4_/KH_2_PO_4_ pH 8.0, 0.05 M NaCl, 0.02 M arginine) and finally for 6–12 hours in (20 mM K_2_HPO_4_/KH_2_PO_4_ pH 7.4, 0.05 M NaCl, 0.05 M arginine). Refolded protein was concentrated with a 10 kDa MWCO centrifugal device (Pall Corporation) at 6000 rpm and 4°C and then stored at -80°C for further analysis.

### On-Column refolding by immobilized metal affinity chromatography (IMAC)

On column refolding was initially carried out as described in the AKTA Prime plus manual and by applying the default on-column refolding program on the chromatography system (GE Lifesciences). However, in order to obtain higher yields of pure r-SgChiC, the method was modified to enhance refolding and elution of refolded protein. 5 different experiments consisting of varying combinations of urea/NaCl concentrations in both refolding and elution buffers (RB and EB) were tested. Details of the combinations are given in supplementary materials and methods (S1 Table). Briefly, the solubilized and reduced r-SgChiC was loaded on a 5 mL His-Trap column equilibrated with 5 CV of BB (20 mM Tris-HCl pH 8, 0.3 M NaCl, 20 mM imidazole and 6 M Gdn-HCl) at a flow rate of 2 mL/min. Following this, a washing step with 5–10 CVs of SB (20 mM Tris-HCl pH 8, 0.3 M NaCl, 20 mM imidazole and 6 M urea) at a flow rate of 1 mL /min was performed. The gradient refolding step was then initiated with 4 CV from 6 M urea in the SB to 0, 1, 2 or 3 M urea in RB (20 mM Tris-HCl pH 8, 0.1–0.5 M NaCl, 20 mM imidazole) at an optimum flow rate of 0.5 mL/min. The protein was washed with 5 CV of 0, 1, 2 or 3 M urea and eluted on a gradient with EB (20 mM Tris-HCl (pH 8), 0.1, 0.3 or 0.5 M NaCl and 0.6 M imidazole) containing 0 to 1 M urea at a flow rate of 0.2 mL/min. Eluted r-SgChiC collected in 2 mL fractions were pooled. The concentration of the pooled peak fractions was adjusted to approximately 0.5 mg/mL by dilution in the ratio 1:3 (buffer to protein) with DB (20 mM K_2_HPO_4_/KH_2_PO_4_ pH 8, 0.05 M NaCl, 0.02 M arginine) supplemented with additional 0.2 M L-arginine as described above. Dialysis, concentration and storage of the refolded protein were subsequently performed as described above.

### Determination of protein concentration, molecular weight and conformation

Protein concentration was determined by Bradford assay using bovine serum albumin (BSA) as standard [33] as well as at 280 nm using an estimated molar extinction coefficient (ε molar) of 66725 M^-1^ cm^-1^. The molecular weight of refolded r-SgChiC was estimated on 12% SDS-PAGE using the method of Laemmli [34] while non-reducing gel electrophoresis was performed by a modified method of Laemmli [34] to observe the various conformations of refolded r-SgChiC. All steps were performed following the traditional SDS-PAGE method except that samples were prepared with sample buffer lacking SDS and β-mercaptoethanol, and without boiling.

### r-SgChiC secondary structure analysis by circular dichroism

Samples obtained from the optimized reverse dilution and on-column refolding methods were buffer exchanged into 10 mM K_2_HPO_4_/KH_2_PO_4_ buffer (pH 8) buffer by dialysis at 4°C for 24 hrs. Circular dichroism (CD) spectra were acquired with a J-815 spectropolarimeter (Jasco) in a 1 mm pathlength cell from 190 to 250 nm with a step size of 0.1 nm and a bandwidth of 1 nm. All measurements were performed at 25°C under a nitrogen flow. The photomultiplier voltage did not exceed 600 V within the required spectral regions. Each spectrum was averaged from three measurements. CD spectrum of the appropriate buffer was recorded and subtracted from the protein spectra. To calculate the secondary structure content of refolded SgChiC, all CD data were analyzed by using the Beta Structure Selection (BeStSel) algorithm web based server [35].

### Assay of refolded enzyme

Enzyme assays were carried out by a modified method of Yagishita and Imoto [36] using colloidal chitin as substrate. Colloidal chitin preparation was modified from Roberts and Selintrennikoff [37]. Purified r-SgChiC solution was added into 0.1 M K_2_HPO_4_/KH_2_PO_4_ buffer to a final concentration of 0.5 nmol and then mixed with 200 μL of 10% (w/v) of moist colloidal chitin suspended in distilled water, in a total reaction volume of 1 mL. The mixture was incubated at 37°C for 30 minutes with agitation and reaction was stopped by boiling for 5 minutes. Residual colloidal chitin was removed by centrifugation at room temperature. The supernatant obtained was mixed with Schale’s reagent (0.5 M Na_2_CO_3_ solution and 0.05% of potassium ferricyanide K_3_[Fe(CN)_6_]) in the ratio of 1:1 and boiled for 15 minutes. The mixture was brought to cooling at room temperature before reading its absorbance at 420 nm. All experiments were carried out in triplicates at least 3–4 times per refolding method. One unit of enzyme activity was defined as the amount of enzyme in mg that released 1 *μ*mol of N-acetyl-D-glucosamine per minute at 37°C.

## Results

### Expression and solubilization of SgChiC inclusion bodies

**Fig 1:**
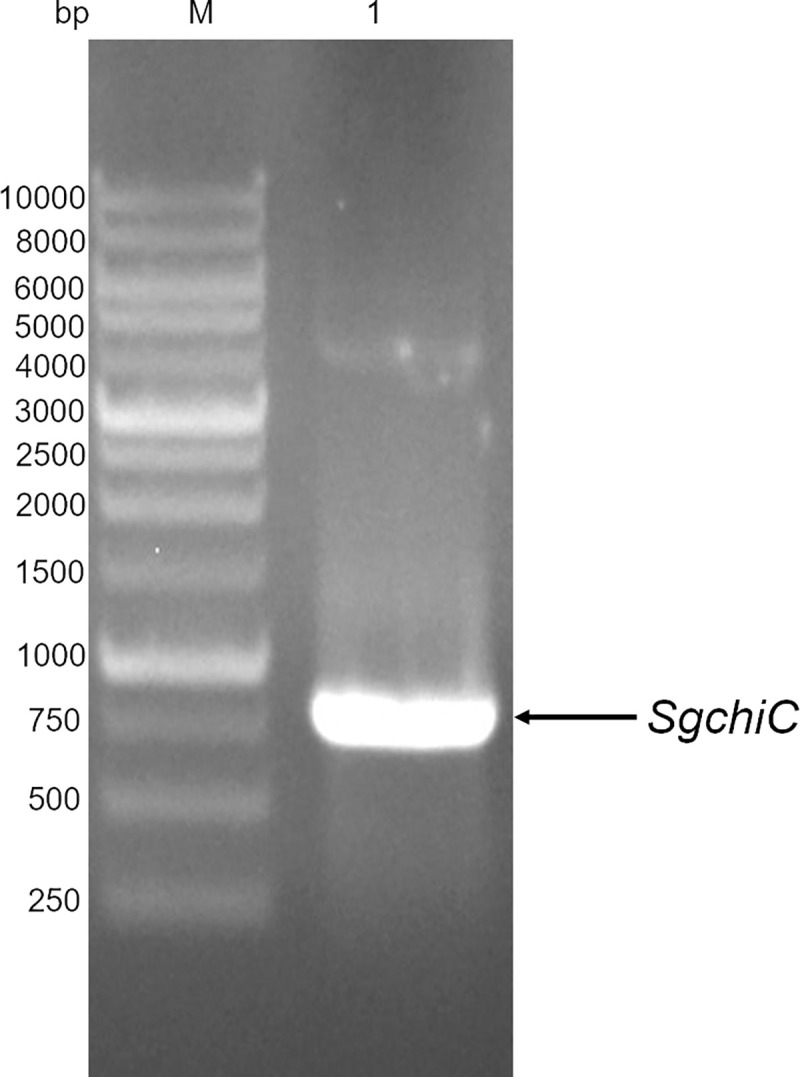
Amplified DNA fragment of the 795 kb insert of *SgChiC* on a 0.8% agarose gel. Lane M. DNA ladder (DM 3100 Excelband 1kb ladder), lane 1. amplified *SgChiC*.

*SgChiC* with codons optimized for expression in *E*. *coli* BL21(DE3) was commercially synthesized and cloned into a pET22-b(+) vector via its *Nde*I-*Xho*I restriction sites. Fig 1 shows the amplified DNA fragment of *SgChiC*. The *pel*B leader peptide was excluded from the expression construct of r-SgChiC (S1 Fig) to prevent deposition of protein in the periplasmic space.

**Fig 2:**
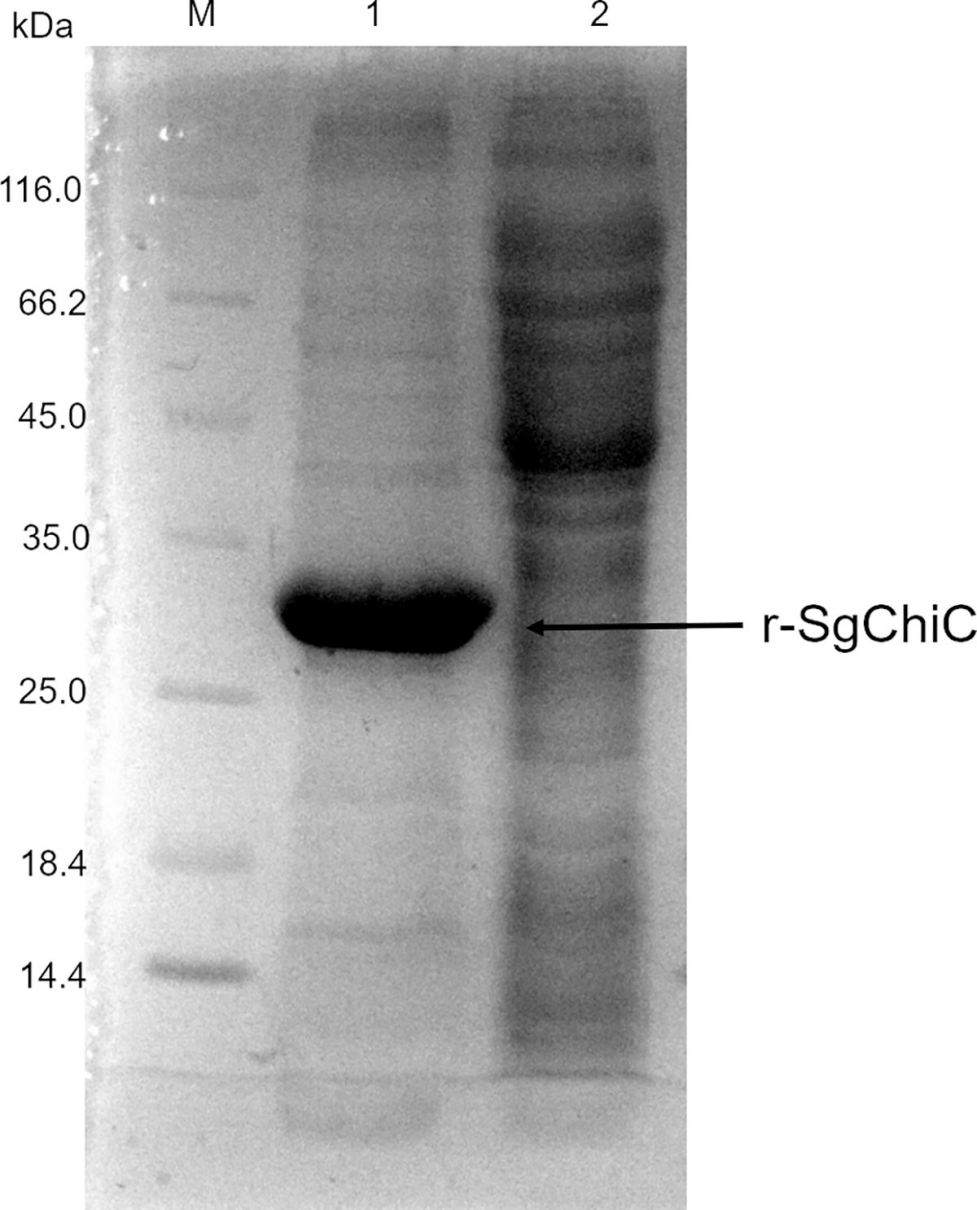
SDS-PAGE analysis of the washed r-SgChiC IBs and clarified lysate of *E*. *coli* BL21(DE3). Lane M, Thermoscientific Pierce unstained molecular weight protein marker. Lane 1, washed IB. Lane 2, clarified lysate.

Recombinant *E*. *coli* BL21(DE3) cells, upon induction with 0.2 mM IPTG at 30°C for 2 hours, overexpressed the C-terminal His-tagged r-SgChiC exclusively as inclusion bodies. The IB fraction harvested after separating the crude lysate was washed 2 times prior to solubilization with wash buffer (50 mM Tris-HCl pH 8, 0.3 M NaCl) supplemented with 1 M urea (Fig 2). No distinct band corresponding to the size of the protein of interest (≈29 kDa, 6-HisTag inclusive) was observed in the clarified cytoplasmic extract after disruption of cells as well as in the supernatants from the washing steps (Fig 2 and S2 Fig).

The solubility of r-SgChiC IB was predetermined by analyzing the turbidity of IB pellets resuspended in different concentrations of Gdn-HCl (3–7 M) at 340 nm (S3 Fig). r-SgChiC IBs were found to be optimally soluble in 6 M Gdn-HCl. Similarly, buffers were screened for their ability to suppress aggregation. The best buffer that resulted in the lowest amount of precipitation as measured at 340 nm contained 0.2 M arginine (S4 Fig). Other additives such as glycerol as well as reduced and oxidized glutathione (GSH/GSSG) when used in RB without arginine did not seem to contribute much to suppressing the formation of aggregates. Therefore, 6 M Gdn-HCl and refolding buffers containing arginine were selected to solubilize and complete refolding of r-SgChiC respectively in subsequent experiments.

### r-SgChiC refolded by reverse dilution (RD)

**Fig 3:**
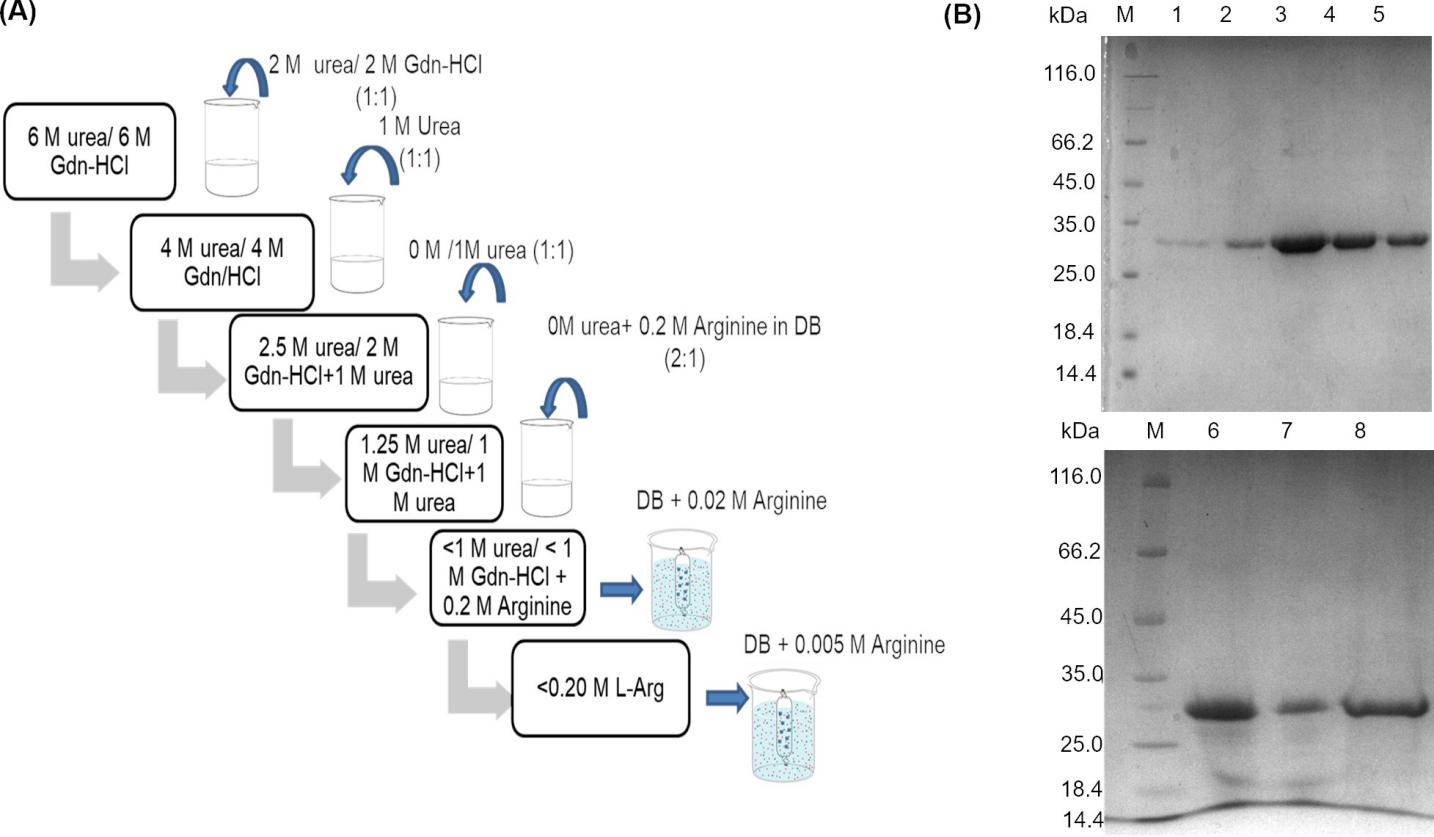
Analysis and schematic representation of RD refolded method. A. Stepwise reverse dilution method used for the refolding of purified protein in 6 M urea or crude protein in 6 M Gdn-HCl respectively. Unfolded protein suspended in high denaturant concentration is gradually introduced to buffer with low concentration of denaturant by dilution of sample with an equal volume of the respective buffer as shown. Excess denaturant is excluded by dialysis in buffer without denaturant but with arginine to suppress aggregation. B. Gel images represent purified r-SgChiC protein before and after refolding by reverse dilution. Gel on the top shows SDS-PAGE analysis of r-SgChiC purified under denaturing conditions with 6 M urea. M—unstained molecular weight marker. Lanes 1–5—peak fractions of the 6-His tagged recombinant SgChiC eluted on a gradient of 0.6 M imidazole in the presence of 6 M urea. Lane 6—refolded sample before dialysis, Lane 7—dialyzed r-SgChiC. Lane 8—concentrated r-SgChiC.

The initial refolding protocols attempted for refolding r-SgChiC were the traditional dilution and dialysis methods as described by [38,39]. These methods resulted in extremely diluted protein and significant precipitation respectively. Afterwards, a refolding protocol involving 4 steps of reverse dilution and 2 steps of dialysis (Fig 3A) was developed and optimized for high yields of r-SgChiC. SDS-PAGE images of purified and refolded fractions are shown in Fig 3B. This method yielded up to 3 times more actively folded purified protein from a 500 mL culture than previous attempts. An average of 12 and 8 mg of r-SgChiC were obtained before dialysis, and after dialysis and concentration respectively (Table 1). These samples recorded specific activities of 28 and 28.8 U/mg respectively.

**Table 1:** Recovery table of r-SgChiC by RD.

Step	Total protein (mg)	Activity (U/ml)	Total Activity (U)	Specific activity (U/mg)	Yield %	Fold
Washed IB	nd	4	nd	nd	nd	-
Solubilized	27	26.6	119.7	4.4	100	1
RD	12	5.6	336	28	280	6.4
Dialyzed and concentrated	8	28.6	228.8	28.6	191	6.5

nd- not determined

### Effects of varying combinations of urea and NaCl on yield of On-column (OnC) refolding r-SgChiC

**Fig 4:**
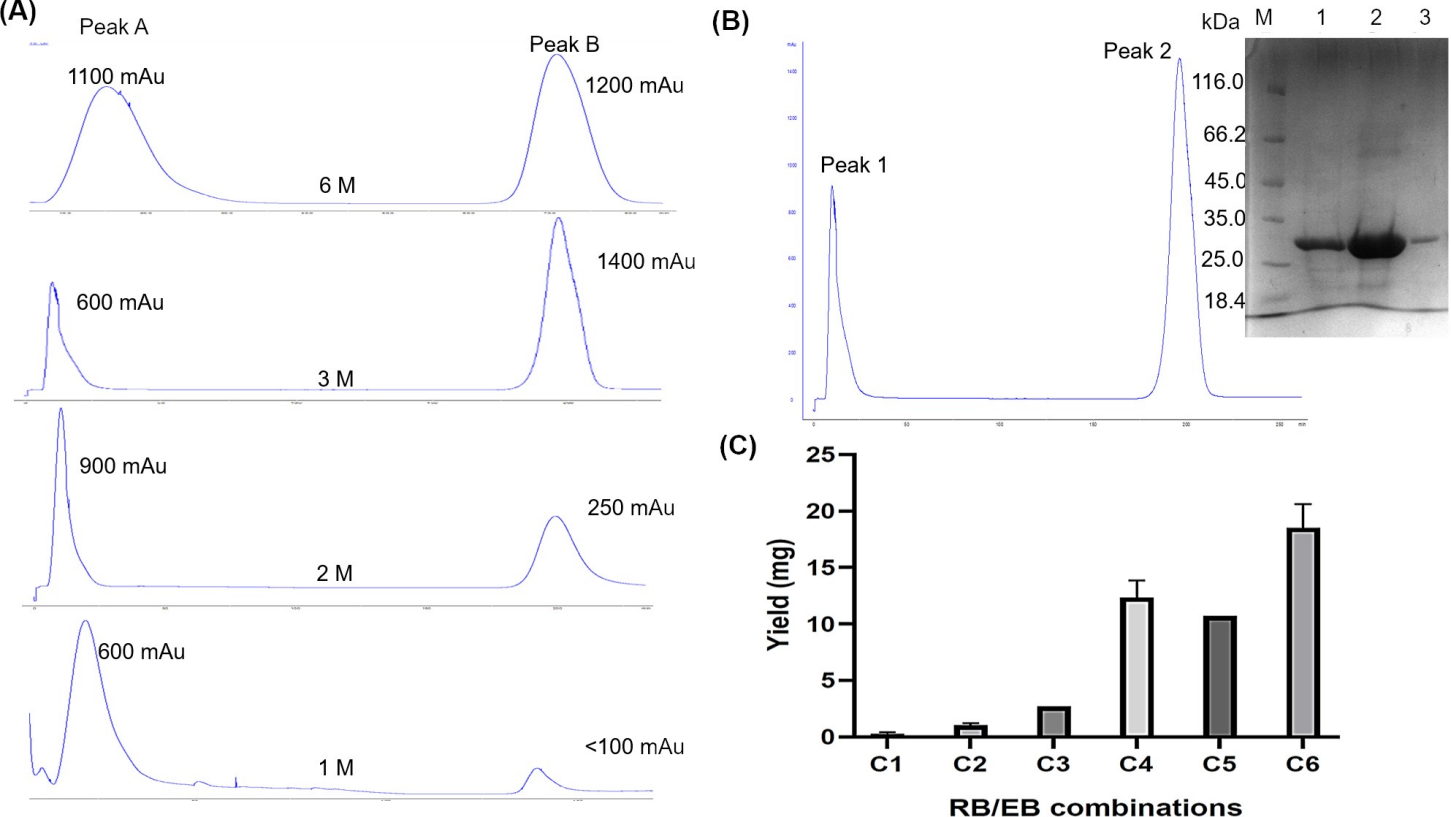
Effects of varying urea and NaCl combinations in RB and EB on yield of OnC refolded r-SgChiC. A. Chromatograms of OnC refolded r-SgChiC with different urea concentrations in the RB. B. Chromatogram of OnC refolded r-SgChiC with buffer C4 (RB/EB containing 3 M urea / 1 M urea, 0.3 M NaCl). Peaks 1 and 2 are the flow through and eluate respectively. Gel image shows SDS-PAGE analysis of peak 2. Lane M—molecular weight marker. Lanes 1–3 represent peak fractions. C. Effects of different combinations of urea and NaCl on the yield of OnC refolded r-SgChiC. **C1**–0 M urea, 0.5 M NaCl in both RB and EB. **C2–**1 M urea, 0.5 M NaCl in RB and EB. **C3—**RB/EB- 2 M/1 M urea, 0.3 M NaCl in both RB and EB. **C4—**RB/EB: 3 M urea/ 1 M urea, 0.3 M NaCl in both RB and EB. **C5—**RB/EB: 3 M urea/0 M urea, 0.1 M NaCl in both RB and EB. **C6—**RB/EB: 3 M urea/ 1 M urea, 0.3 M NaCl, 2 mM GSH, 0.3 mM GSSG in both RB and EB.

The on-column refolding method carried out by applying the standard AKTA Prime Plus programme and recommended buffer conditions yielded less than 1 mg of purified protein from a 500 mL culture. The yield remained the same even when the culture volume was increased to 1 L. In order to optimize yield, on-column refolding was carried out under varying urea and NaCl combinations in RB and EB respectively (S2 Table). Sodium chloride concentration was reduced from 0.5 M to 0.3 M in buffers C3 and C4 and to 0.1 M in C5 to observe if the increase in yield was due to reduction of NaCl. However, there was no corresponding increase in yield with low NaCl concentration in C5. The difference in the yields observed could not be attributed to changes in NaCl concentration. Instead, an increasing yield of protein was detected during elution with increasing urea (0–3 M) in RB (Fig 4A). The optimum yield was obtained with buffer C4 with RB/EB combination of 3 M/1 M urea both supplemented with 0.3 M NaCl respectively (Fig 4B). This was comparable to the yield detected under denaturing conditions with 6 M urea as shown in the chromatograms (Fig 4A). The concentrated protein after a 2-step dialysis of the pooled peak fraction yielded approximately 8 mg. Buffer C6 containing the redox couple GSH/GSSG equally resulted in high yields of r-SgChiC up to 20 mg (Fig 4C). However, this buffer was considered unsuitable due to continuous protein precipitation in subsequent steps and during storage.

### Effects of refolding method and buffer composition on oligomeric state of r-SgChiC and activity

As observed in all reducing SDS-PAGE gels, samples refolded OnC and by dilution yielded single bands (Figs 3B and 4B) of ~29 kDa molecular weight. Non-reducing PAGE also revealed high concentrations of the monomeric forms although with extra bands of dimers (~58 kDa) and multimers (~116 kDa) (S5 Fig). Based on the structural study of SgChiC the most preferred form of the protein is the monomeric form compared to its other forms [2]. The dimers and multimers are more noticeable in OnC refolded samples due to their high yield compared to sample refolded by dilution as a result of its dilute state after dialysis. Since the redox pair appeared not to have any distinct impact on the yield of well folded r-SgChiC, the buffer combination C4 without GSH/GSSG was used for subsequent assays and analysis. r-SgChiC refolded using buffer C4 recorded a specific activity of 52.3 U/mg (Table 2).

**Table 2:** Purification/recovery table of r-SgChiC refolded on-column by affinity chromatography.

Step	Total protein (mg)	Activity (U/ml)	Total Activity (U)	Specific activity (U/mg)	Yield %	Fold
Washed IB	nd	nd	nd	nd	nd	nd
Solubilized	27	26.6	119.7	4. 4	100	1
OnC refolded	16	56.7	567.0	29.8	473.6	6.8
Dialyzed and concentrated	8	74.6	596.8	52.3	498.5	11.8

nd- not determined

Although this was 2 times higher than the RD refolded r-SgChiC, both OnC and RD refolded samples in this study resulted in comparable specific activities as reported previously with SgChiC extracted and purified by osmotic shock (Table 3).

**Table 3:** Comparison of yield and specific activities of refolded and native OnC SgChiC.

Cellular Component	Recovery	Culture volume (mL)	Yield (mg)	Specific activity (U/mg)	Reference
Inclusion body	Refolded RD	500	8–12	28	This study
Inclusion body	Refolded OnC	500	8–12	52.3	This study
Periplasmic (native)	Osmotic shock	1000	10	24.5	[32,40]

### Secondary structure prediction of refolded r-SgChiC using circular dichroism spectroscopy

**Fig 5:**
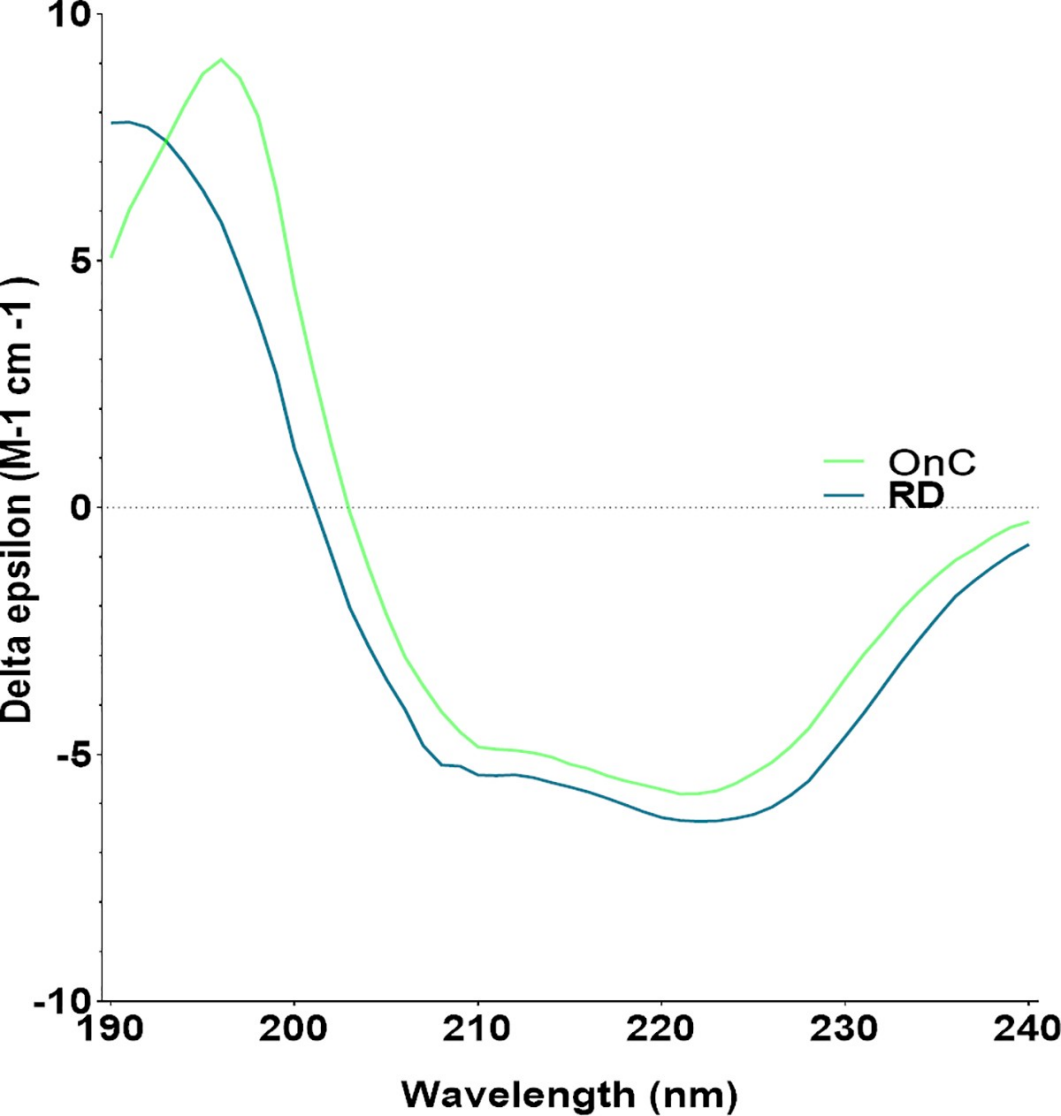
Circular dichroism spectra obtained for both dilution and on-column refolded samples.

Circular dichroism spectra calculated for r-SgChiC samples refolded by reverse dilution and on-column chromatography revealed similar trends with slight deviations in the detection signal at 190–195 nm and at 210–240 nm (Fig 5).

Both refolding methods showed closely related secondary structure components with the presence of positive bands forming helices and beta sheets between 190 and 195 nm and; negative bands at 209 and 222 nm respectively. Helices and beta sheets were estimated to be at 38.4 and 2.2% respectively for RD sample; and 43 and 6% for OnC refolded sample. These results are comparable to the secondary structure component in the crystal structure (PDB ID: 1WVU, 2DBT and 2D49) [26] with 39–41% helices and 7% beta sheets as estimated with DSSP [41].

## Discussion

The expression of r-SgChiC exclusively as inclusion bodies even after optimizing conditions of expression such as temperature, induction time and IPTG concentration necessitated the development of a refolding protocol. Similarly, in previous studies, initial attempts to express the protein in an *E*. *coli* JM109 host, resulted in extremely low or no yields [32]. Subsequently, pET12a and pET22b were used for the overexpression of the enzyme in the periplasm of *E*. *coli* BL21(DE3) with yields up to 10 mg from 1 L culture [32,40]. These vectors encode signal peptides (OmpT and pelB respectively). When fused to the gene of interest, it traverses the cytoplasmic membrane via the sec translocon pathway and directs the localization of protein in the periplasmic space [42]. Proteins with disulphides are reported to fold correctly in the periplasm because of the oxidizing environment and the presence of foldases which facilitate correct formation of disulphides [43–47]. Upon localization in the periplasm of *E*. *coil* BL21(DE3), SgChiC was retrieved in previous studies by the cold osmotic shock method and further purified by a series of steps including ammonium sulphate precipitation and chromatography on a hydroxyapatite column [27,32,40,47]. Here, the pET22-b(+)::*SgchiC* expression construct was designed with and without the leader peptide for a possible periplasmic and cytoplasmic expression respectively. However, SgChiC was not detected in the periplasmic fraction of *E*. *coli* BL21(DE3)::pET-*SgChiC* construct (with the pelB leader peptide). On the contrary, analysis of cell lysates of the construct without the pelB construct revealed sufficient yields of protein equivalent to the molecular weight of SgChiC in the IB fraction. Therefore, the expression system without the pelB signal peptide, subsequently referred to as pET22-b(+)::*SgchiC* was retained and used continuously for the expression of r-SgChiC throughout this study. r-SgChiC IB was subsequently subjected to different refolding conditions. Finally, actively folded r-SgChiC was recovered by a reverse dilution and an on-column affinity chromatography method.

While developing a refolding routine either on-column or by dilution it was observed that harvesting cells in their early stationary phase resulted in IB with less impurities. Hence post induction growth of *E*. *coli* BL21(DE3) cells harbouring pET22-b(+)::*SgchiC* was terminated after a period of only 2 hours at 30°C. IBs isolated at much later periods did not result in significantly higher yields but posed a greater challenge during solubilization. A similar observation was reported in a previous study whereby the solubility of inclusion bodies harvested within 2 hours of post induction resulted in higher solubility than those isolated beyond 2 hours [48]. Another important observation was that the washing step enhanced the recovery of purer forms of IB (Fig 2) with less background proteins especially when refolding the crude protein directly by dilution without prior purification steps. Washing the pellets with 1 M urea as described in an earlier report [39] partially purified the pellets and reduced endogenous *E*. *coli* proteins that were soluble or that may have accumulated with r-SgChiC as inclusion bodies. This eliminated the need for the use of detergents like Triton X-100 which would otherwise have required further wash steps especially before on-column refolding can be performed as described elsewhere [1,10].

r-SgChiC was optimally solubilized with 6 M Gdn-HCl in 50 mM Tris-HCl buffer, pH8 as determined in a preliminary experiment (S3 Fig). r-SgChiC IB could be classified as classical since it was resistant to solubilization under mild conditions and lower concentrations (3, 4 and 5 M) of Guanidine-HCl as observed from the initial buffer screening (S3 Fig). Solubilizing at such low concentrations rapidly formed significant precipitation upon introduction of refolding buffer. Classical IBs are described as tough, dense, resistant to protease attack and soluble only in high concentrations of denaturants [48] while non-classical IB are reported to have high amounts of correctly folded target proteins which could be expressed at lower temperatures or solubilized under mild denaturing conditions.

The initial refolding trials in this study resulted in either extremely low yields or overly diluted protein suspensions. This was because r-SgChiC has a high propensity for aggregation during refolding even at low concentrations as it readily precipitated under medium to low denaturing buffer conditions during dilution, on-column refolding and dialysis (S6A Fig). Aggregation is considered the most difficult and frequently encountered problem during any refolding process. This is attributed to: 1) the formation of mismatched or non-native intra and intermolecular interactions related to the number of cysteine residues; 2) the hydrophobic nature of unfolded proteins which tend to collapse into non-native states under low denaturant concentrations. Hence, refolding conditions are designed to inhibit protein aggregation. This is best achieved when the protein concentration is kept as low as possible, hence the principle of refolding by dilution in large volumes of buffer [4,8].

In the RD method applied here, each step was optimized to suppress aggregation as well as to achieve high yields with the lowest possible volume of buffer. The gradual reduction of denaturant (Gdn-HCl) from 6 M to < 1 M therefore took place in four steps of reverse dilution (Fig 3A). The buffer used in all steps was supplemented with some amount of urea rather than making use of buffer with no denaturant. Washed IB was first solubilized and reduced in 20 mM Tris-HCl, pH 8, 300 mM NaCl supplemented with 6 M Gdn-HCl and 10 mM DTT. The solubilized sample was either purified under denaturing conditions before refolding or directly refolded by reverse dilution. In the first dilution step, the protein sample was diluted 2 times with the same buffer as above, supplemented with 2 M urea or Gdn-HCl to give 3M Gdn-HCl in the presence of urea or 4 M Gdn-HCl respectively, as modified from a previously described method [10]. Subsequent refolding steps were achieved by further dilution as shown in Fig 3A. The substitution of Gdn-HCl with urea during the stepwise dilution was to reduce its ionic effect on the refolded enzyme as well as to stabilize r-SgChiC during dialysis. Urea is known to have a more subtle effect on proteins compared to Gdn-HCl and believed to stabilize refolded protein at low concentrations [49]. Applying the reverse dilution method in this study enabled the ease of further processing by dialysis and ultracentrifugation. This is because an initial volume of 5 mL with up to 6 mg/mL of protein could be diluted about 10-fold with little or no significant formation of aggregates. The final volume of protein suspension obtained (approximately 50 or 60 mL) was easily subjected to oxidative refolding by dialysis in the presence of arginine, and then subsequently concentrated to the desired volumes.

Refolding by dilution has always been the easiest and most practiced method for the refolding of solubilized IBs. However, the conventional method of dilution requires the addition of proteins into large volumes of buffers (at least 100-fold) leading to extremely dilute samples. This renders further down-stream processing and retrieval of protein in concentrated forms very difficult. Other disadvantages of refolding by conventional dilution has been clearly reported [3,9], hence, the choice of a reverse dilution method in this study. Another advantage of reverse dilution method over the conventional dilution is that the refolding process and chemicals are controlled in terms of volume, denaturant concentration and flow rates of buffers added into the sample [7]. In addition, since the dilution process was stepwise in the of presence of intermediate to low concentration of denaturant, refolding took place gradually and any observed precipitates could be easily filtered out or excluded by centrifugation before proceeding to the next step.

**Fig 6:**
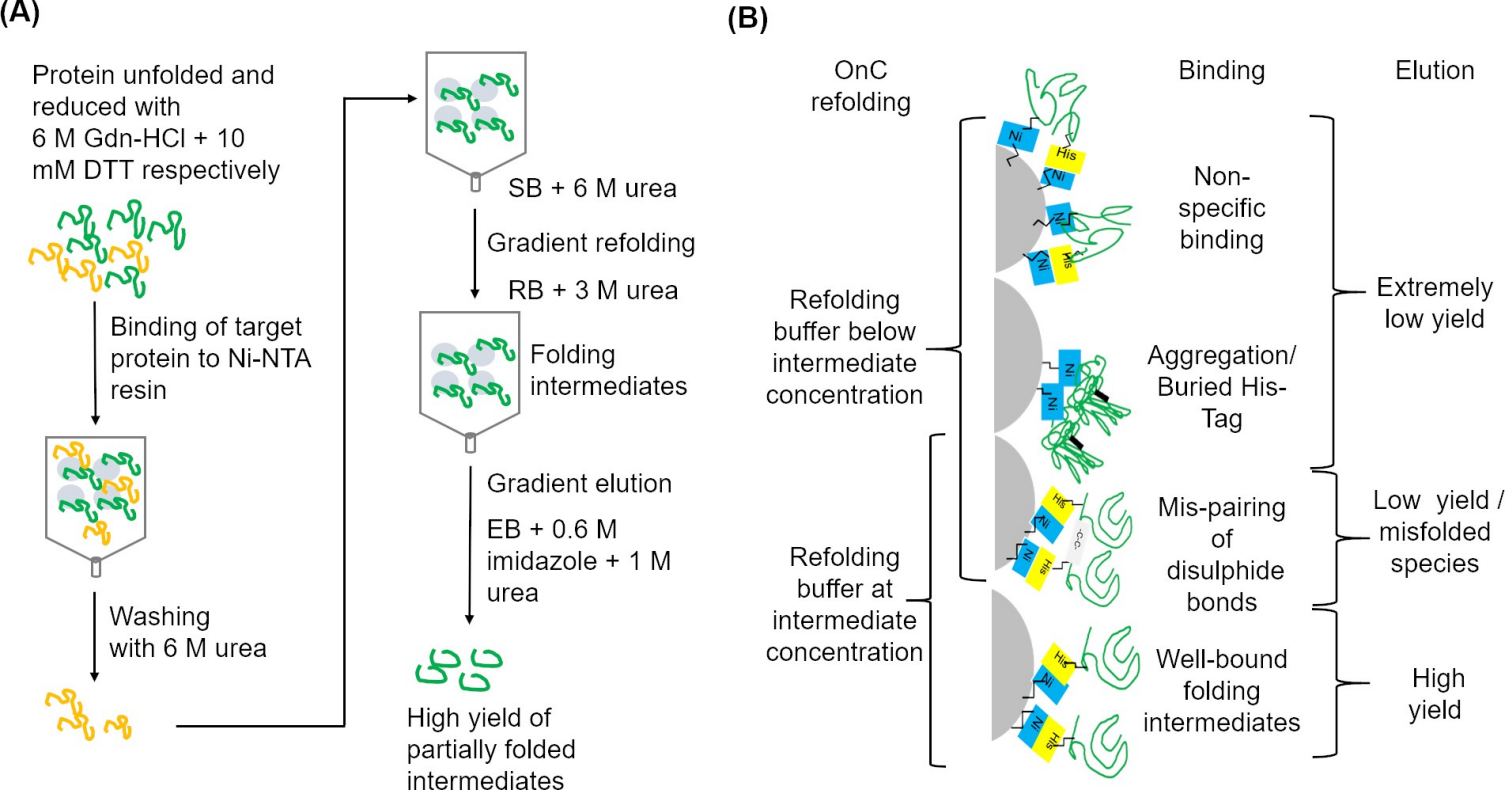
Schematic representation of on-column refolding method. A. Representation of binding, gradient refolding in intermediate concentration of urea in RB and final gradient elution with low urea concentration B. Representation of the possible binding conformations of r-SgChiC on Ni-NTA column.

The principle of refolding on-column is similar to reverse dilution since buffer is added to sample in both cases except that refolding takes place with the protein bound to a matrix in the on-column method. Additionally, on-column refolding has the advantage of simultaneous purification of the sample, hence it can be completed within a relatively shorter period than first purifying under denaturing conditions before refolding by reverse dilution. Initial direct application of the recommended protocol and default parameters in the AKTA PRIME plus system in this study however resulted in extremely low protein yields. The low yields were attributed to one or all of the following: on-column aggregation resulting from incorrect folding or partially folded intermediates; inefficient elution resulting from a firm hydrophobic attachment of refolded protein to the resin; accumulation of proteins at the upper part of the column resulting in precipitation and eventual loss in yield as highlighted earlier [24]. Hence the refolding protocol in this study was optimized to address these challenges. Some attempts made include switching columns from 1 mL to 5 mL and increasing the flow rate for loading the enzyme onto the 5 mL His-Trap column from 0 to 2 or 3 mL per minute. Bound protein molecules were subsequently washed, refolded and eluted at lower flow rates of 1, 0.5 and 0.2 mL/ min respectively. In previous trials with a 1 mL His-trap column using similar sample volumes (5 mL), it appeared that unfolded proteins in the crude material possibly interacted hydrophobically with each other and with the surface of the resin which in turn contributed to their stacking at the upper part of the column. This stacking was observed as a discolored patch that remained even after washing and elution. We propose that the larger surface area of the 5 mL column as well as higher flow rate during sample loading probably reduced the stacking of solubilized proteins with exposed hydrophobic residues at the upper part of the column. This enhanced the rapid distribution of unfolded proteins (up to 25 mg) throughout the resin during binding. Consequently, the usage cycle and efficiency of the column was maintained. A similar observation was made by Langenhof et al. [50] who reported the reduction in the recovery of protein with increased protein load on an ion-exchange column. The study attributed low yield to the poor spatial isolation of protein molecules on the matrix that consequently promoted aggregation of incompletely refolded protein. In some studies, protein samples were loaded and bound to resins in batches supposedly to avoid the phenomenon of stacking [51–53]. On-column aggregation in another study was suppressed by washing columns extensively with detergents [52]. While these methods were successful in those studies, they are time consuming. Our study sought to increase yield and complete the refolding within a short time, as such the method described here encouraged the high yield of partially folded intermediates which were subjected to final refolding conditions during dialysis. Lower flow rates especially during the gradient refolding and elution steps may have contributed to enhancement in the yield of refolded r-SgChiC as it was slowly exposed to the buffers with lower concentration of urea. In an earlier report by Gu et al. [6], factors such as lower flow rates at 0.1 and 0.5 mL /min in a 5 mL His-trap chelating column during the course of refolding along a linear gradient yielded well folded proteins of higher yields compared to flow rates of 1 and 5 mL/min. Similar situations were observed in this study as a 1 mL/min flow rate during the gradient refolding step resulted in lower yields compared to a flow rate of 0.5 mL/min. Additionally, a low urea concentration of 3 and 1 M added into refolding and elution buffers respectively enhanced the elution step. The refolding buffer with 3 M urea served as an intermediate denaturing concentration that stabilized and maintained the binding of protein to nickel by the His-tag (Fig 6A). Lower urea concentrations in the refolding buffer resulted in poor elution as r-SgChiC remained bound to the column. The possible ways by which His-tagged r-SgChiC remained bound to the column is illustrated in Fig 6B. Thus, the presence of 3 M urea slowed down the refolding process thereby reducing the hydrophobic attachment of protein to column. The incidence of on-column aggregation and formation of unfavorable inter and intra molecular disulphide bonds were also suppressed. Elution was then commenced on a gradient and urea concentration reduced to about 1 M (Fig 6A). 1 M urea in the eluate, just as in the reverse dilution method was retained to maintain the stability of r-SgChiC in subsequent dialysis steps. The function of low urea concentration has been reported elsewhere as having a stabilizing effect at low concentrations on proteins being refolded [5,54,55]. Varying the concentration of urea in the refolding buffer enabled the manipulation of the binding of r-SgChiC which consequently influenced the efficiency of elution and protein yield (Fig 6B).

Dialysis was performed as a necessary step for the removal of excess salts and denaturant. This further enhanced the refolding yield (Tables 1 and 2). To complete the refolding, the eluted r-SgChiC sample was dialyzed in buffer supplemented with 0.05 M arginine. Prior to dialysis, fractions containing eluted r-SgChiC were pooled and diluted in the ratio of 1:2 with the final refolding buffer supplemented with additional arginine (0.2 M). It was observed that complete refolding of SgChiC was optimal at high pH above 8.0. The alkaline condition was a result of the addition of arginine in dialysis buffer. The pH of the dialysis buffer in the presence of arginine was raised from 8.0 to 10–10.5. Without the addition of arginine, dialyzed protein aggregated in a short time. Also, the effect of arginine in suppressing protein aggregation was lost when buffer was titrated back to lower pH levels.

It appears therefore that an alkaline condition of pH 8 and above was more suitable for the efficiency of L-arginine in suppressing aggregation during dialysis. Burgess et al., [24] also noted that refolding of proteins generally takes place under alkaline conditions of pH 8 and above. Gu et al. [8], achieved refolding of *E*. *coli* IBs by gel filtration along with an increasing pH gradient from 2 to 7.5. The study suggested that pH and urea gradient were important for correct refolding. A recent study combined the use of high hydrostatic pressure under alkaline pH in the presence of arginine to refold a recombinant dengue 1 virus NSI IB and found out that high yields were obtained at a high pH of 11 and a slightly higher volumetric yield at a pH of 10.5 in the presence of arginine [21]. In a separate study, a recently developed systematic protein refolding screen method incorporated arginine as an essential “helper” additive in refolding of the receptor binding domain of hemagglutinin (HA-RBD) [56].

This observation provides evidence for the stabilizing effect of arginine on enzymes. Although the mechanism for this is not yet clear, it appears that the basic and hydrophilic nature of arginine enhances solubility by acting as a non-denaturing solvent which interacts with hydrophobic patches in proteins [54,57,58]. This possibly reduces the interaction of exposed residues to solvent and surfaces. A direct interaction of exposed hydrophobic regions of proteins with buffer components may initiate rapid motions of unfolded or partially folded intermediates resulting in unfavorable inter and intramolecular interactions thereby causing the protein to collapse into a misfolded state. However, it is worthy to note that arginine is non-denaturing and does not act like other chaotropic agents [9,54]. From observations made in this study, arginine at concentrations up to 0.2 M or more may be capable of bringing IB into solution without perturbing the conformations of the molecules which may be in their unfolded, partially folded, misfolded or aggregated states.

The success of the refolding methods developed in this study was achieved by suppressing unfavorable interactions at every stage. Aggregation is suppressed in traditional dilution by adding aliquots of protein directly or in pulses into large volumes of buffers with very little or no denaturant [4,59] while in the on-column method, refolding takes place on a gradient of 6 to 0 M urea i.e. during the transition from a denaturing condition to a low or non-existent one. These principles did not favor refolding of r-SgChiC and resulted in significant aggregation. Hence, the optimized reverse dilution and on-column refolding methods relied on an intermediate denaturant concentration (3 M) to achieve high yields of refolded r-SgChiC (S6B Fig). The intermediate denaturant concentration seemed to sufficiently maintain solubility while the refolding process was slowly initiated and before final removal in subsequent steps. This suggests that solubilized proteins have a critical point of solubility in the presence of chemical and physical agents at which refolding can be initiated. Hence, we recommend that for every refolding trial, this critical point can be determined to attain optimum yield and efficiency. Mild refolding conditions involving the use of detergents [12], reversed micelles, and an intermediate or low concentration of denaturant [60] as well as physical conditions such as alkaline pH and/or high hydrostatic pressure have been reported [16,21,22,61] as strategies for high yields. HHP refolding has particularly been used successfully to refold several proteins including an oligomeric [17] as well as a disulphide rich protein [15] both of which demonstrated reasonably high yields of well folded protein. Such yields are obtainable since solubilization takes place without disrupting hydrogen bonds that maintain their secondary structure. Under such conditions, protein molecules that have native-like structures remain intact and are easily recovered [2]. However, high pressure refolding requires specialized equipment which may not always be readily available [23]. Also, just like every other refolding method, the development of a working refolding protocol has to go through series of trials and optimization processes [24]. Conventional refolding by RD and OnC methods make use of routine protein purification equipment and therefore can be readily applied to any initial laboratory scale refolding process for the purpose of biochemical and structural studies. Although, both methods resulted in similar yields, the RD method is more time consuming especially if purification under denaturing conditions is performed prior to reverse dilution. The OnC refolding is however desirable in that 2 processes (refolding and purification) can be achieved in a single step. In general, both methods are very simple and straight forward requiring few chemical agents and can be a first trial step for the refolding of any protein expressed as IB. This is the first report that describes an alternative and efficient means for obtaining r-SgChiC from inclusion bodies rather than by the cold osmotic shock method. It is also one of few recent reports describing the optimized refolding of IB by reverse dilution and affinity chromatography.

## Supporting information

S1 Fig Plasmid maps showing pET22-b(+) only and pET22-b::*SgChi*C. (TIF)

S2 Fig SDS-PAGE visualization of solubilized r-SgChiC and supernatants from washing steps. M Protein molecular weight marker. Lane 1, r-SgChiC. Lanes 2 and 3 are supernatants from 2 washing steps.
(TIF)

S3 Fig Screening of buffers supplemented with varying concentration of Gdn-HCl (3–7 M) for the solubility of r-SgChiC. Low absorbance at 340 nm indicates less aggregation.
(TIF)

S4 Fig Screening of buffer additives for the refolding of r-SgChiC. **Solubilized protein is diluted in the ratio 1:10 and the absorbance at 340 nm.** Low absorbance indicates better refolding and less aggregation.
(TIF)

S5 Fig Non-reducing gel electrophoresis of refolded r-SgChiC. M—molecular weight marker, Lane 1—Onc refolded sample with buffer C4, C6 and C5 respectively, Lane 4—Reverse dilution refolded sample.
(TIF)

S6 Fig Schematic representation of refolding by traditional dilution in comparison with stepwise refolding in this study. (A) Previous method whereby protein is refolded in buffer containing very low concentration of denaturant. This resulted in loss of protein due to aggregation when applied to the refolding of r-SgChiC in this study (B) Refolding by reduction of denaturant from high–intermediate–low concentrations. Refolding is afterwards completed by dialysis, during which all residual denaturant is excluded from the protein.
(TIF)

S1 Table Refolding buffers screened for the suppression of aggregates. (PDF)

S2 Table Refolding and elution buffer combinations examined for the high yield of OnC refolded r-SgChiC. (PDF)

S1 File (PDF)
