# Supplementary figures and images for: Effective refolding of a cysteine rich glycoside hydrolase family 19 recombinant chitinase from Streptomyces griseus by reverse dilution and affinity chromatography
Source: PLoS One. 2020 Oct 22;15(10):e0241074. doi: 10.1371/journal.pone.0241074 (PMC7580917; doi:10.1371/journal.pone.0241074)

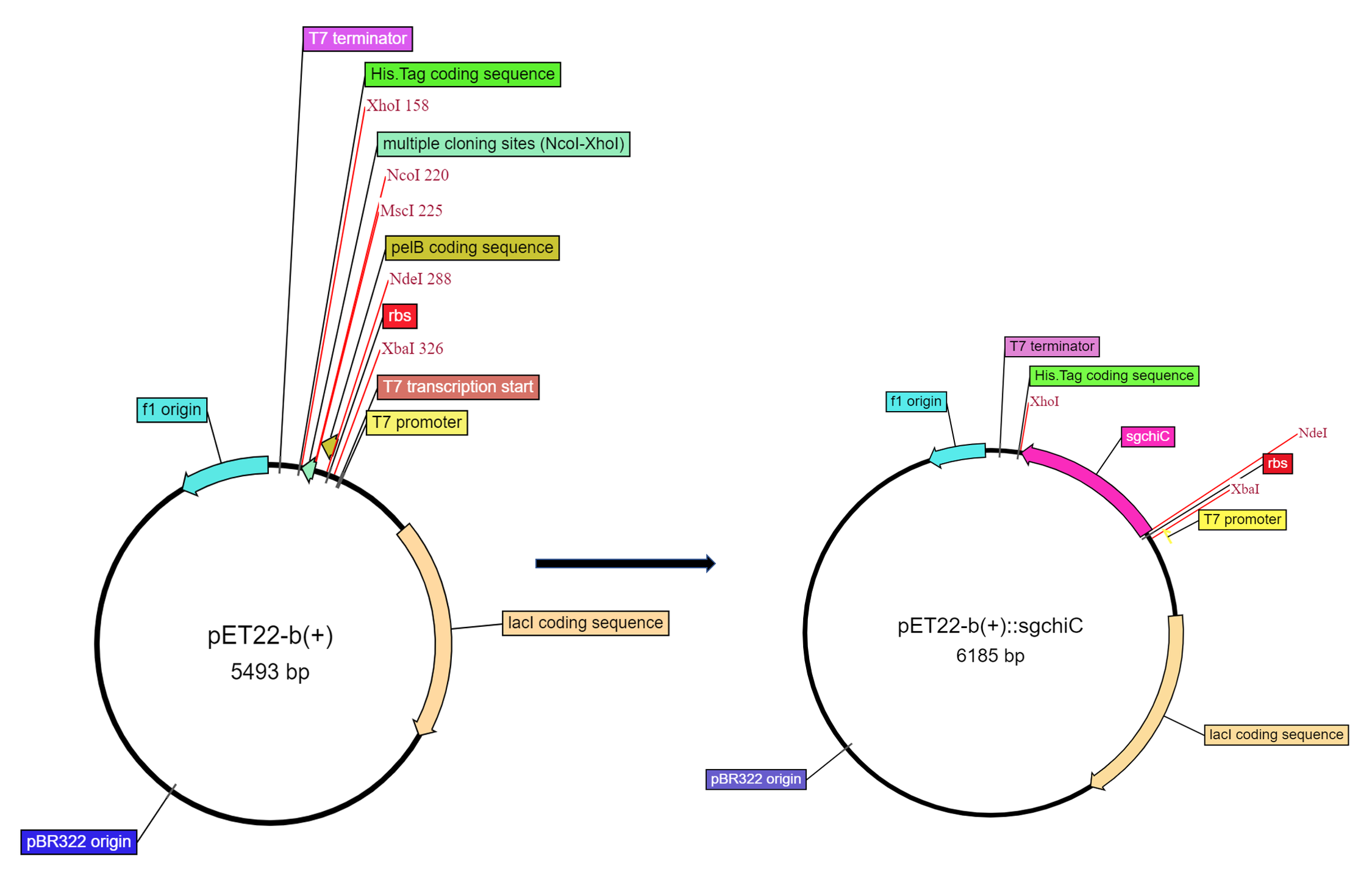

Supplement: S1 Fig — (TIF) [file pone.0241074.s001.tif]

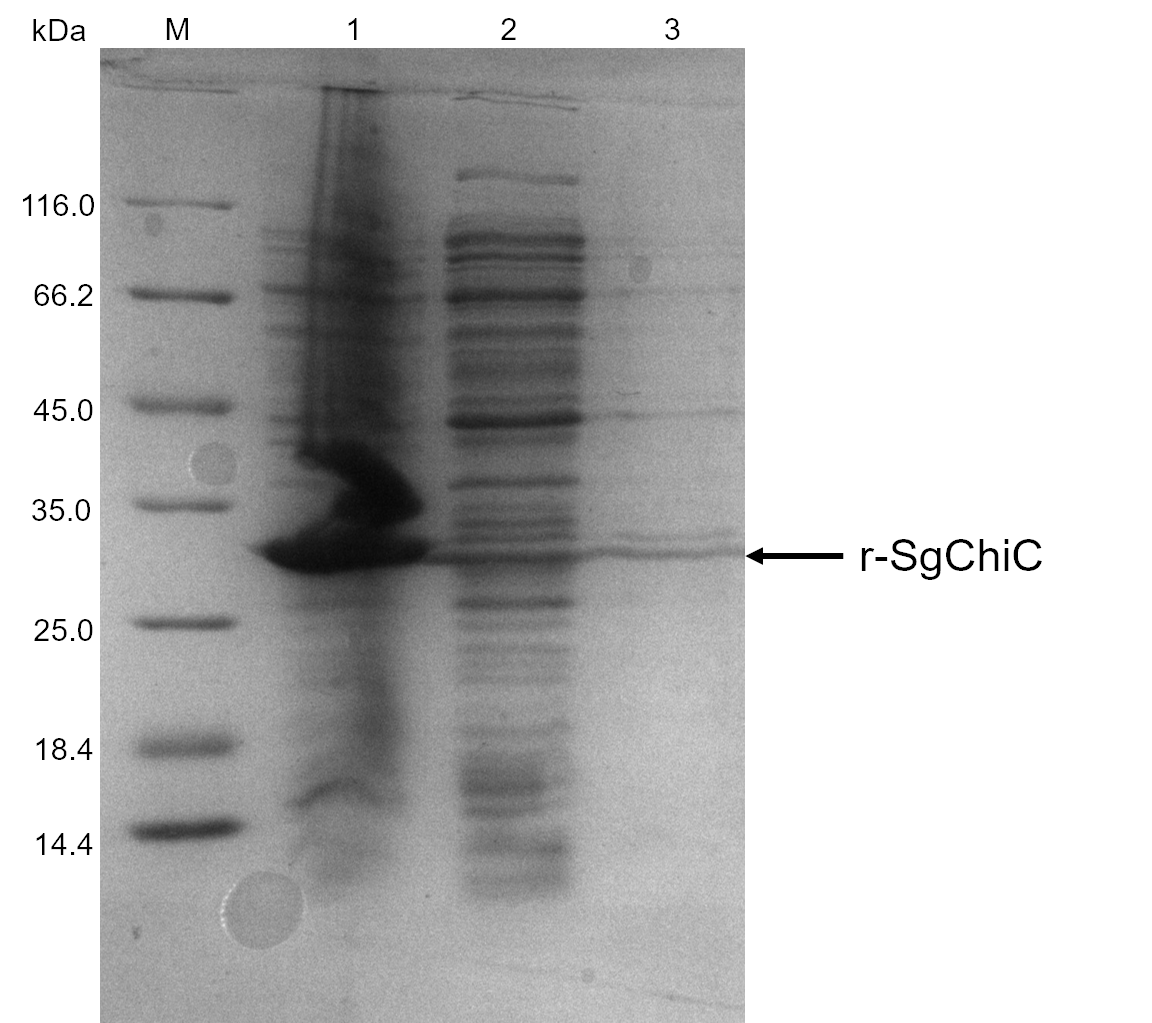

Supplement: S2 Fig — M Protein molecular weight marker. Lane 1, r-SgChiC. Lanes 2 and 3 are supernatants from 2 washing steps. (TIF) [file pone.0241074.s002.tif]

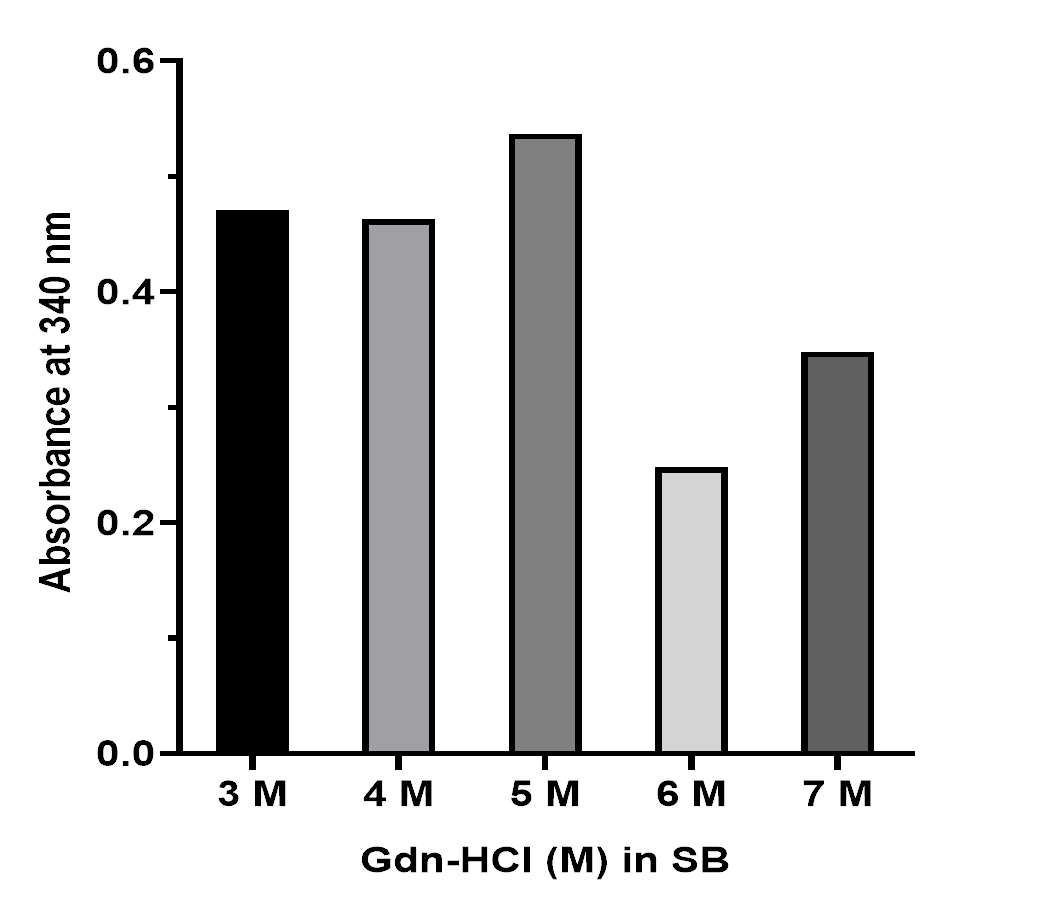

Supplement: S3 Fig — Low absorbance at 340 nm indicates less aggregation. (TIF) [file pone.0241074.s003.tif]

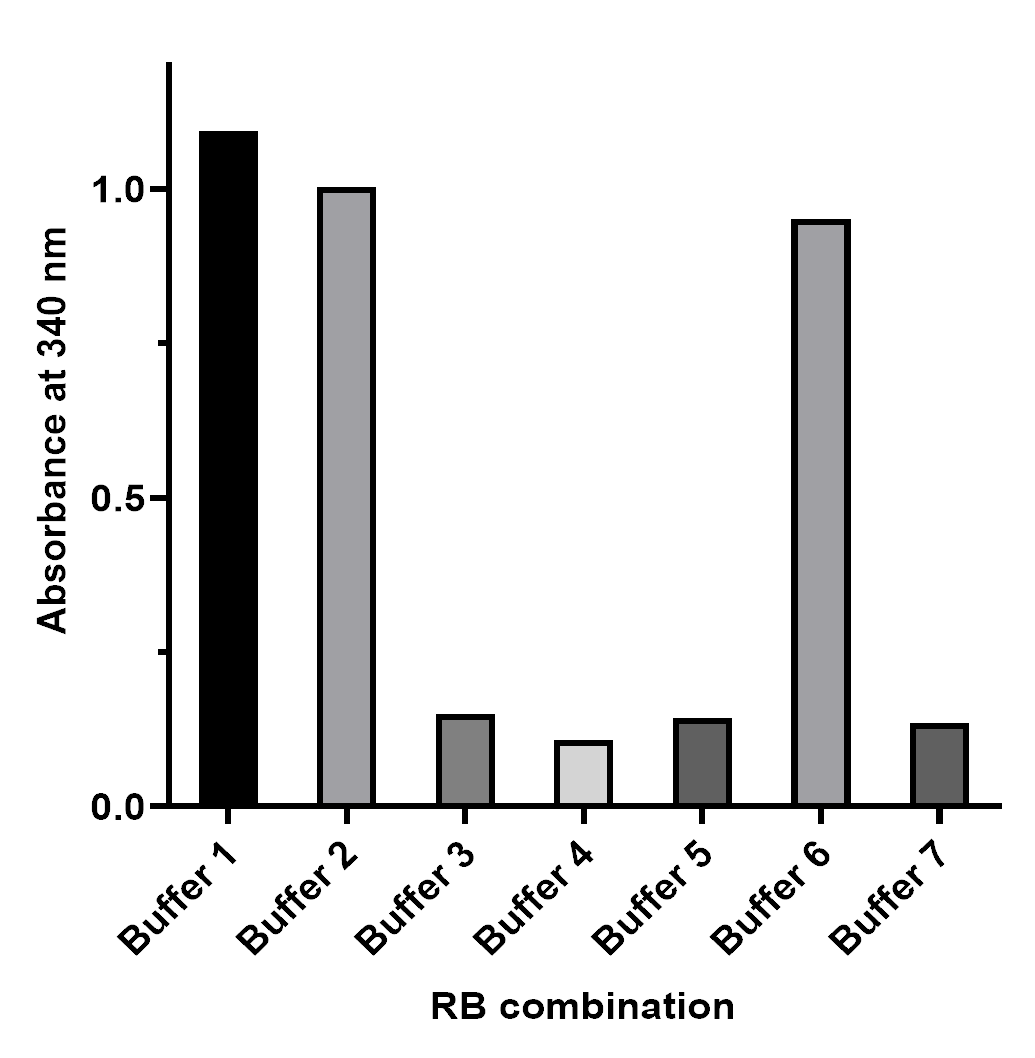

Supplement: S4 Fig — Solubilized protein is diluted in the ratio 1:10 and the absorbance at 340 nm. Low absorbance indicates better refolding and less aggregation. (TIF) [file pone.0241074.s004.tif]

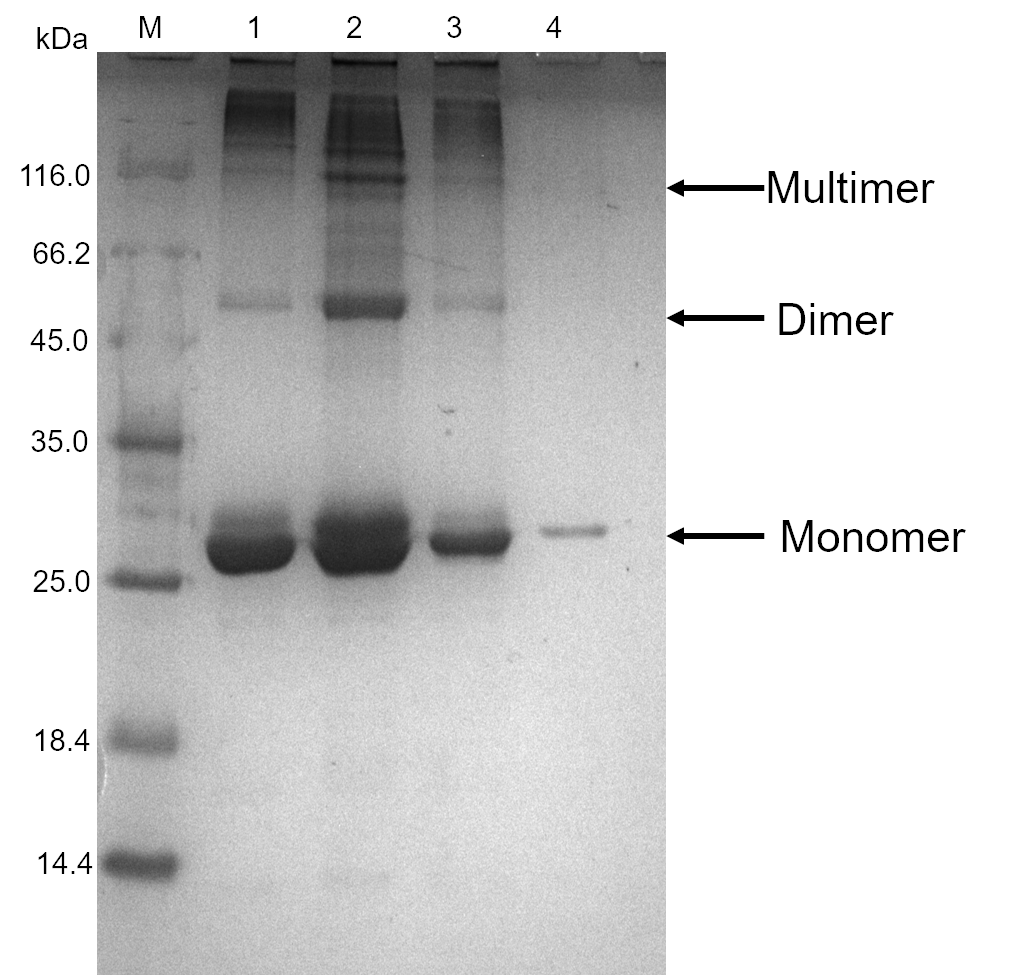

Supplement: S5 Fig — M—molecular weight marker, Lane 1—Onc refolded sample with buffer C4, C6 and C5 respectively, Lane 4—Reverse dilution refolded sample. (TIF) [file pone.0241074.s005.tif]

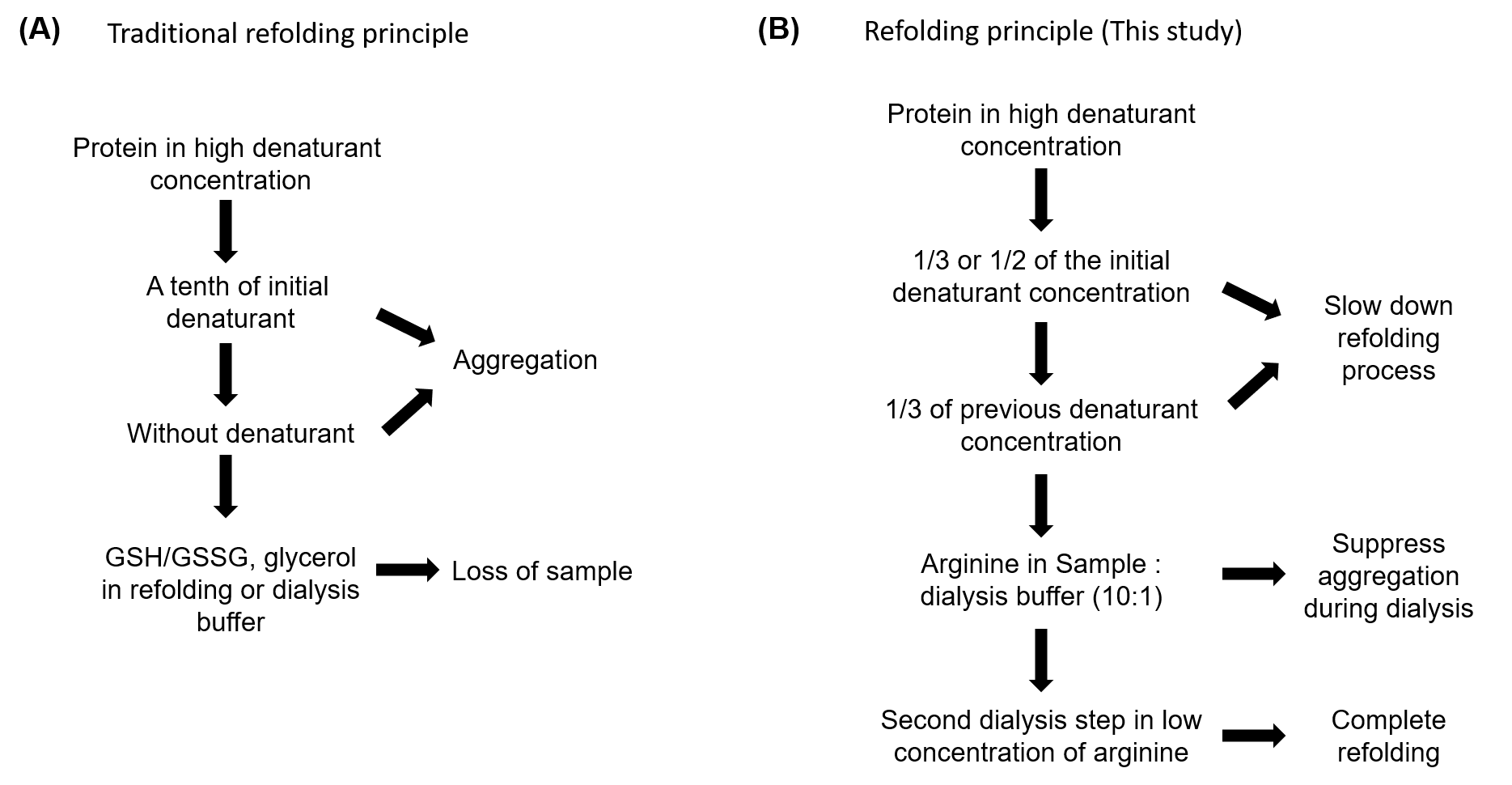

Supplement: S6 Fig — (A) Previous method whereby protein is refolded in buffer containing very low concentration of denaturant. This resulted in loss of protein due to aggregation when applied to the refolding of r-SgChiC in this study (B) Refolding by reduction of denaturant from high–intermediate–low concentrations. Refolding is afterwards completed by dialysis, during which all residual denaturant is excluded from the protein. (TIF) [file pone.0241074.s006.tif]
