# Supplementary material for: Effective refolding of a cysteine rich glycoside hydrolase family 19 recombinant chitinase from Streptomyces griseus by reverse dilution and affinity chromatography
Source: PLoS One. 2020 Oct 22;15(10):e0241074. doi: 10.1371/journal.pone.0241074 (PMC7580917; doi:10.1371/journal.pone.0241074)
Supplement: S1 Table — (PDF) [file pone.0241074.s007.pdf]

**S1 Table:** Initial screening of refolding buffers and additives for the suppression of aggregates

| Buffer | Refolding Buffer + additives |
| --- | --- |
| 1 | RB only |
| 2 | RB + 1 M urea |
| 3 | RB + 1 M + 0.2 M arginine |
| 4 | RB + 0.2 M arginine |
| 5 | RB + 1 M urea + GSSG+ GSH+ 0.2 M arginine |
| 6 | RB + 1 M urea + 5% glycerol |
| 7 | RB + 1 M urea + 0.2 M Arginine + 5% glycerol + GSSG + GSH |

RB - 0.5 M Tris-HCl pH 8, 0.5 M NaCl, 20 mM immidazole
