# Supplementary material for: Effective refolding of a cysteine rich glycoside hydrolase family 19 recombinant chitinase from Streptomyces griseus by reverse dilution and affinity chromatography
Source: PLoS One. 2020 Oct 22;15(10):e0241074. doi: 10.1371/journal.pone.0241074 (PMC7580917; doi:10.1371/journal.pone.0241074)
Supplement: S2 Table — (PDF) [file pone.0241074.s008.pdf]

S2 Table: Refolding and elution buffer combinations examined for the high yield of OnC refolded r-SgChiC

Buffer combinations	Urea (M) in RB/EB	NaCl (M) in RB and EB	GSH/GSSG (mM) in RB/EB
C1	0	0.5	-
C2	1	0.5	-
C3	2/1	0.3	-
C4	3/1	0.3	-
C5	3/0	0.1	-
C6	3/1	0.3	2/0.3
