# Supplementary material for: Effective refolding of a cysteine rich glycoside hydrolase family 19 recombinant chitinase from Streptomyces griseus by reverse dilution and affinity chromatography
Source: PLoS One. 2020 Oct 22;15(10):e0241074. doi: 10.1371/journal.pone.0241074 (PMC7580917; doi:10.1371/journal.pone.0241074)
Supplement: S1 File — (PDF) [file pone.0241074.s009.pdf]

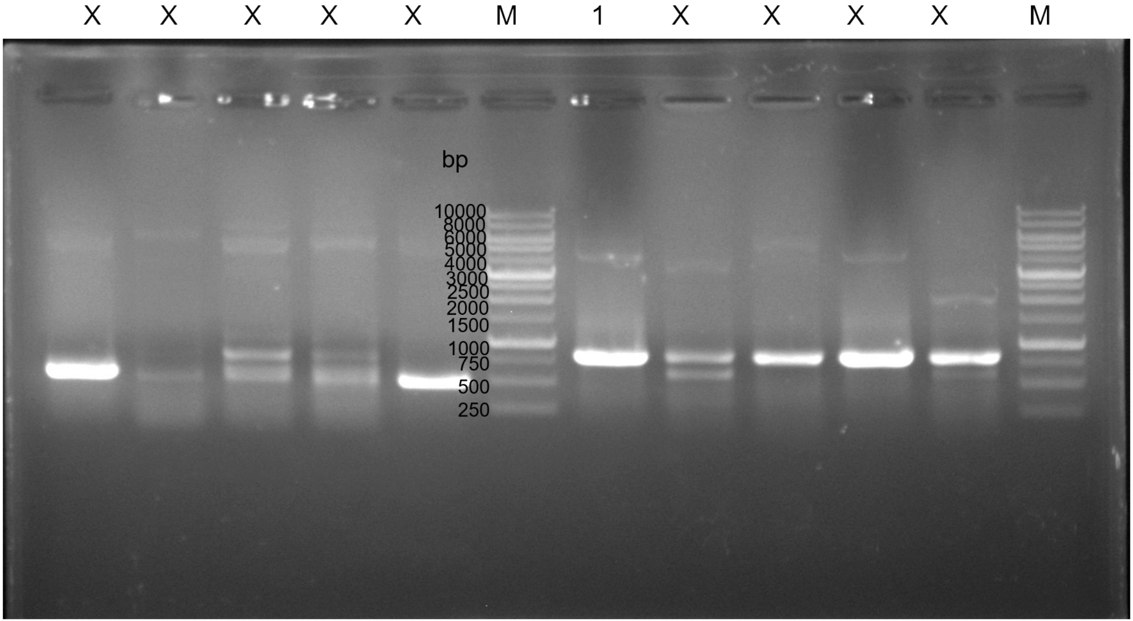

Fig 1. Amplified DNA fragment of the 795 kb insert of *SgchiC* on a 0.8% agarose gel.
Lane M. DNA ladder (DM 3100 Excelband 1kb ladder), lane 1. amplified *SgchiC*

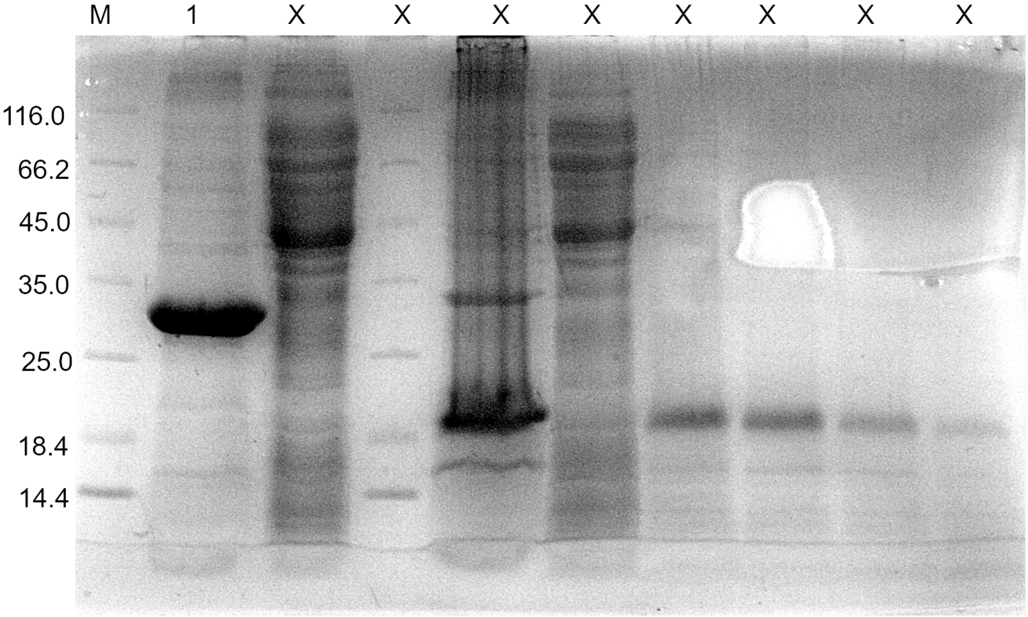

Fig 2. SDS-PAGE analysis of the washed r-SgChiC IBs and clarified lysate of *E. coli* BL21(DE3). Lane M, Thermoscientific Pierce unstained molecular weight protein marker. Lane 1, washed IB. Lane 2, clarified lysate.

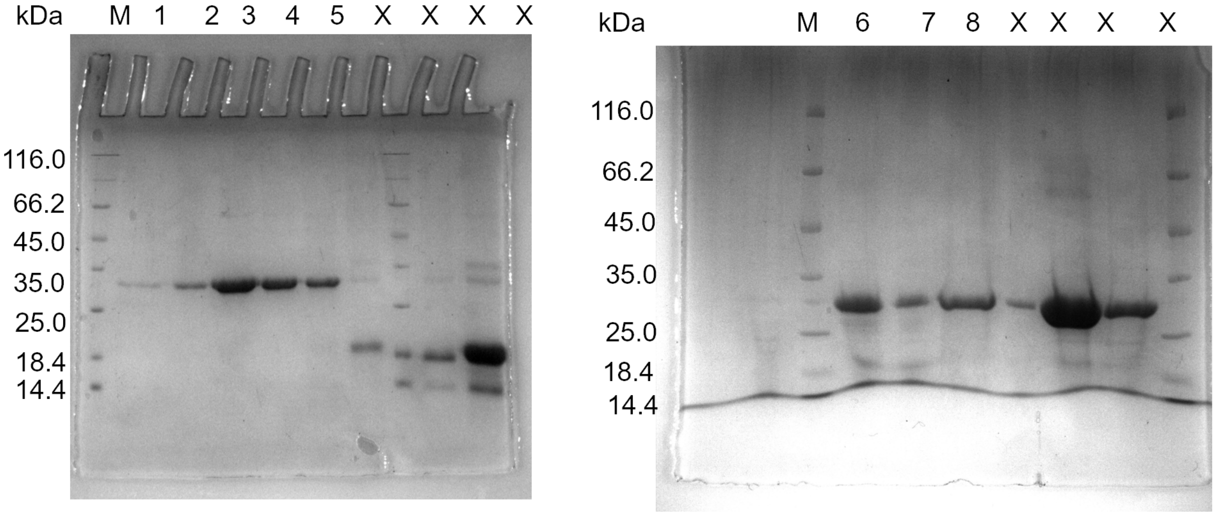

Fig 3. Analysis and schematic representation of RD refolded method. r-SgChiC purified under denaturing conditions with 6 M urea. M - unstained molecular weight marker. Lanes 1-5 - peak fractions of the 6-His tagged recombinant SgChiC eluted on a gradient of 0.6 M imidazole in the presence of 6 M urea. Lane 6 - refolded sample before dialysis, Lane 7 - dialyzed r-SgChiC. Lane 8 - concentrated r-SgChiC.

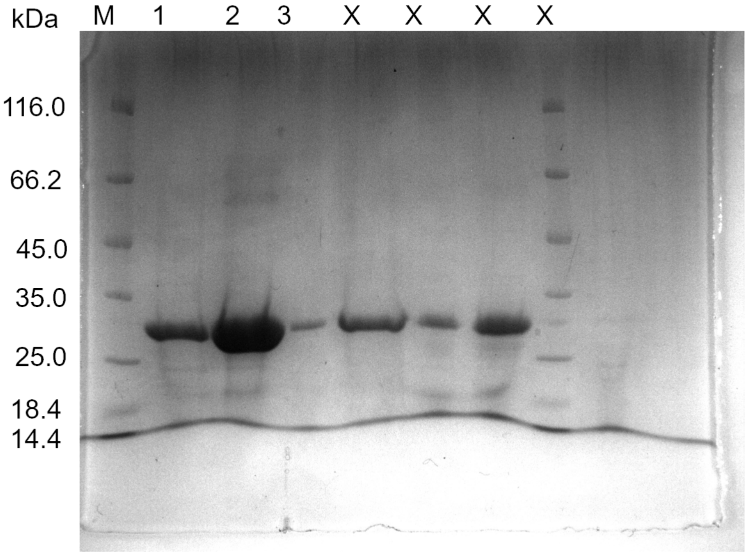

Fig 4. Gel image shows SDS-PAGE analysis of peak 2. Lane M - molecular weight marker. Lanes 1-3 represent peak fractions.

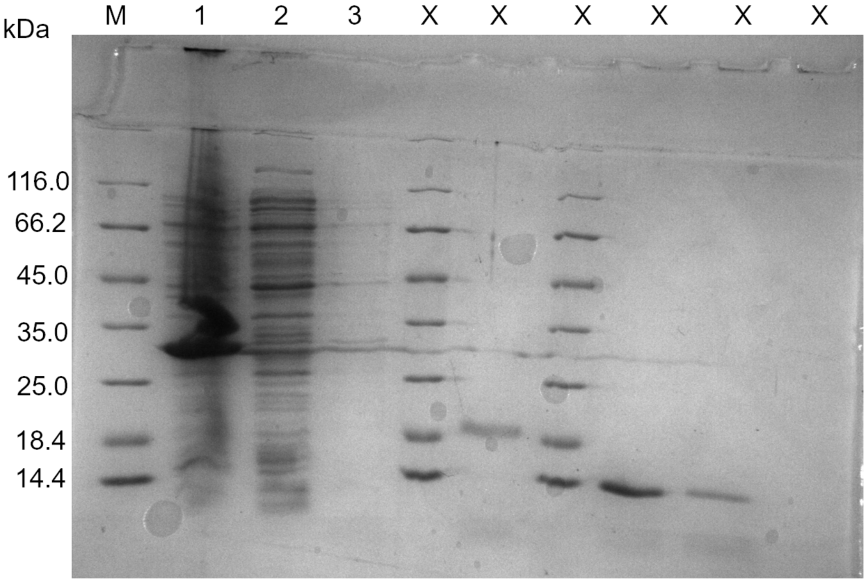

S1 Fig. SDS-PAGE visualization of solubilized r-SgChiC and supernatants from washing steps. M Protein molecular weight marker. Lane 1, r-SgChiC. Lanes 2 and 3 are supernatants from 2 washing steps.

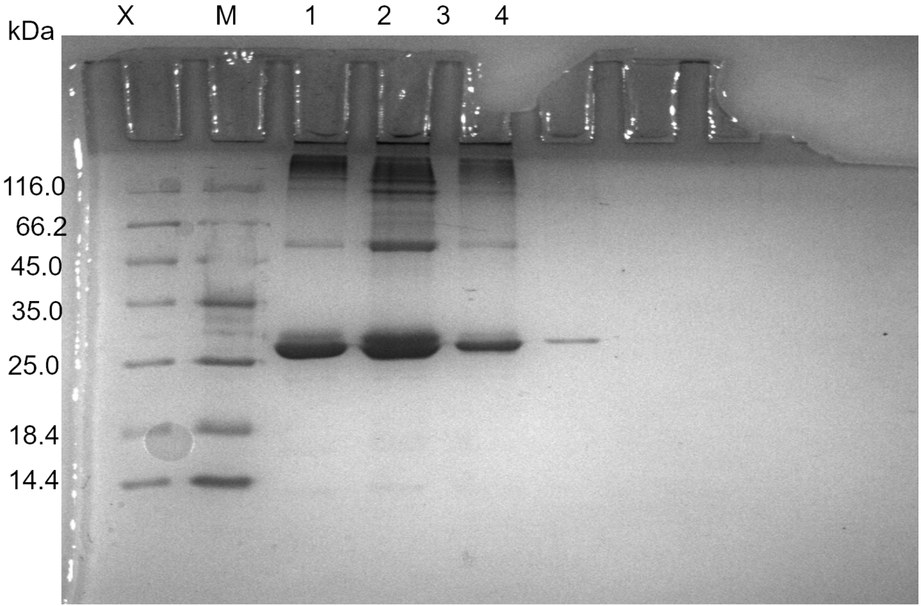

S4 Fig. Non-reducing gel electrophoresis of refolded r-SgChiC . M- molecular weight marker, Lane 1 Onc refolded sample with buffer C4, C6 and C5 respectively, Lane 4- Reverse dilution refolded sample.

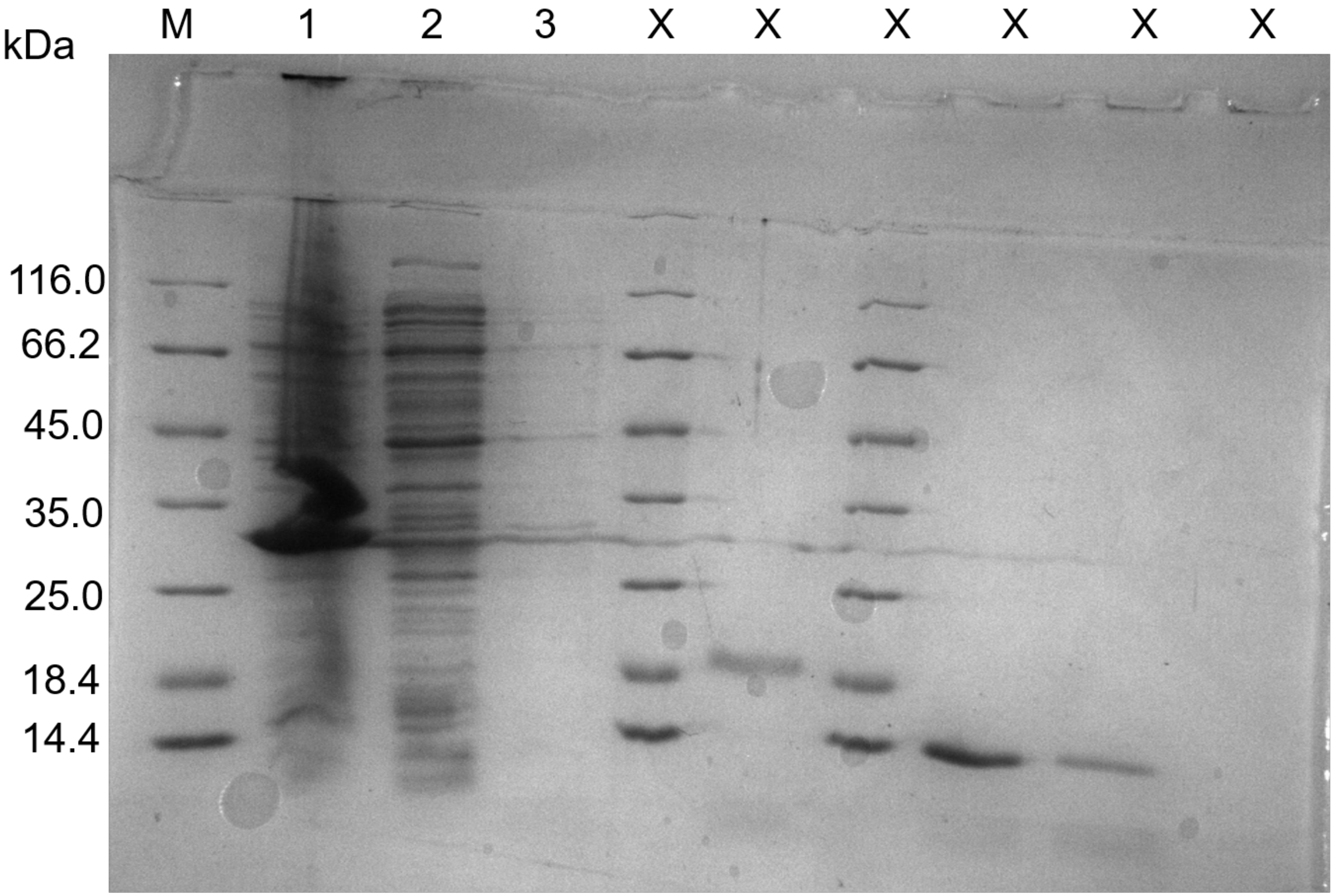

S1 Fig. SDS-PAGE visualization of solubilized r-SgChiC and supernatants from washing steps. M Protein molecular weight marker. Lane 1, r-SgChiC. Lanes 2 and 3 are supernatants from 2 washing steps.
